# Prostaglandin E2 Receptor 4 (EP4) as a Therapeutic Target to Impede Breast Cancer-Associated Angiogenesis and Lymphangiogenesis

**DOI:** 10.3390/cancers13050942

**Published:** 2021-02-24

**Authors:** Guillermo Antonio De Paz Linares, Reid Morgan Opperman, Mousumi Majumder, Peeyush K. Lala

**Affiliations:** 1 Department of Anatomy and Cell Biology, Schulich School of Medicine and Dentistry, University of Western Ontario, London, ON N6A5C1, Canada; gdepazli@uwo.ca; 2 Department of Biology, Brandon University, Brandon, MB R7A6A9, Canada; oppermrm72@brandonu.ca (R.M.O.); majumderm@brandonu.ca (M.M.)

**Keywords:** breast cancer, triple-negative breast cancer, metastasis, angiogenesis, lymphangiogenesis, COX-2, PGE2, EP receptors, EP4, chemokines, cancer stem cells, EMT, microRNAs, patient-derived xenograft (PDX), inflammation, immune checkpoint inhibitors (ICI)s, combination therapy

## Abstract

**Simple Summary:**

The formation of new blood (angiogenesis) and lymphatic (lymphangiogenesis) vessels are major events associated with most epithelial malignancies, including breast cancer. Inflammation is a key mediator of both processes, hijacked by many cancers by the aberrant expression of the inflammation-associated enzyme cyclo-oxygenase (COX)-2. In this review, we focus on breast cancer and show that COX-2 is a major promoter of both events, primarily resulting from the activation of prostaglandin (PG) E receptor EP4 on tumor cells, tumor-infiltrating immune cells, and endothelial cells; and induction of oncogenic microRNAs. The COX-2/EP4 pathway also promotes additional events in breast cancer progression, such as cancer cell migration, invasion, and the stimulation of stem-like cells. Based on a combination of studies using multiple breast cancer models, we show that EP4 antagonists hold a major promise in breast cancer therapy in combination with other modalities including immune check-point inhibitors.

**Abstract:**

The formation of new blood (angiogenesis) and lymphatic (lymphangiogenesis) vessels are major events associated with most epithelial malignancies, including breast cancer. Angiogenesis is essential for cancer cell survival. Lymphangiogenesis is critical in maintaining tumoral interstitial fluid balance and importing tumor-facilitatory immune cells. Both vascular routes also serve as conduits for cancer metastasis. Intratumoral hypoxia promotes both events by stimulating multiple angiogenic/lymphangiogenic growth factors. Studies on tumor-associated lymphangiogenesis and its exploitation for therapy have received less attention from the research community than those on angiogenesis. Inflammation is a key mediator of both processes, hijacked by many cancers by the aberrant expression of the inflammation-associated enzyme cyclo-oxygenase (COX)-2. In this review, we focus on breast cancer and showed that COX-2 is a major promoter of both events, primarily resulting from the activation of prostaglandin (PG) E receptor EP4 on tumor cells, tumor-infiltrating immune cells, and endothelial cells; and the induction of oncogenic microRNAs. The COX-2/EP4 pathway also promotes additional events in breast cancer progression, such as cancer cell migration, invasion, and the stimulation of stem-like cells. Based on a combination of studies using multiple breast cancer models, we show that EP4 antagonists hold a major promise in breast cancer therapy in combination with other modalities including immune check-point inhibitors.

## 1. Introduction

### 1.1. Physiological Roles of Angiogenesis and Lymphangiogenesis

Angiogenesis and lymphangiogenesis are normal physiological processes, important in fetal and post-natal development, tissue homeostasis, and wound repair. Angiogenesis is the process by which new blood vessels are produced from pre-existing vasculature and ensures that every cell in the body has access to adequate oxygen and nutrients [1]. Similarly, lymphangiogenesis is the process by which new lymphatic vessels are formed from pre-existing vessels of the lymphatic system. The lymphatic system is responsible for maintaining the balance of interstitial fluid in the extracellular matrix and allowing the circulation of immune cells (lymphocytes, macrophages, and dendritic cells) throughout the body. A key distinction between these two processes and embryonic vasculogenesis is that in embryonic vasculogenesis, vessels originate *de novo* from vasculogenic precursors called angioblasts within the embryonic mesenchyme.

**Figure 1:**
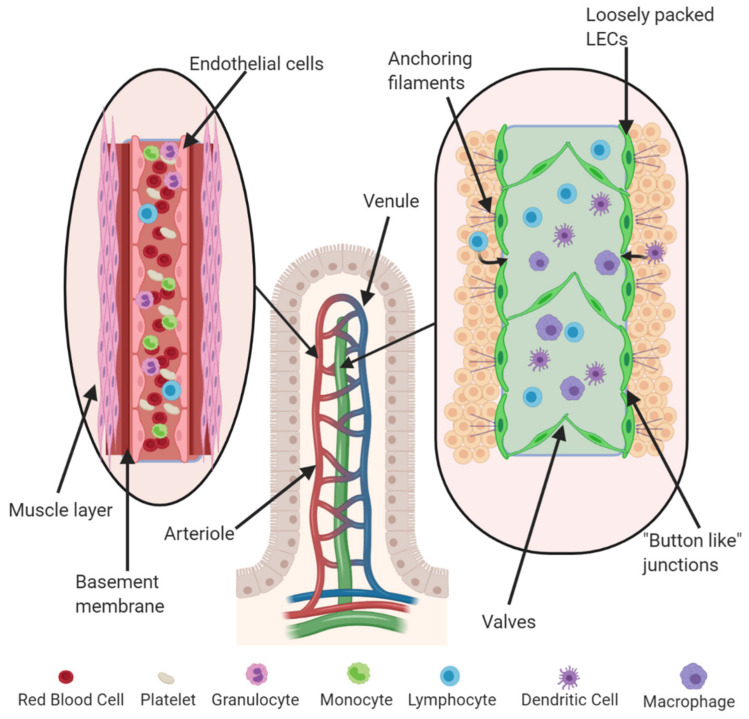
Structure of intestinal villus with associated vasculature and lymphatic vessels. The vascular endothelium loops around from arteries to veins and back to the heart. It contains endothelial cells tightly packed against each other, with an outer layer of smooth muscle cells to facilitate blood flow. Lymphatic vessels are composed of lymphatic endothelial cells (LECs), which are loosely packed to facilitate the exchange of lymph, which is then moved through the vessels by a system of valves. They are connected through “button-like” junctions and are anchored to the extracellular matrix (ECM) by anchoring filaments.

Blood vessels (arteries, arterioles, veins, and venules) are lined by vascular endothelial cells (VECs) surrounded by a layer of smooth muscle cells. Arterioles and venules branch out from larger vessels until they become capillaries lacking in the muscular coat (8–10 µm); these are the smallest blood vessels where oxygen exchange takes place (Figure 1). 

The lympho–vascular network carries the interstitial fluid back to the venous system and permits the recirculation of immune cells. Lymphatic vessels are lined by lymphatic endothelial cells (LECs) starting at the extracellular space as lymphatic capillaries and connect to lymph nodes as afferent lymphatics. Unlike blood capillaries, lymphatic capillaries do not loop back to their starting point, and their leaky walls allow for the collection of lymph, which is then transported using a system of valves found within these vessels. Lymphatic capillaries are nearly three times larger than blood capillaries (10–60 µm in diameter), lined with a single layer of LECs. Unlike blood capillaries, the basal lamina of lymphatic vessels is incomplete, discontinuous, or even absent and lack surrounding pericytes and smooth muscle cells (Figure 1). The majority of inter-endothelial cell interactions are maintained by “button-like” junctions. The nature of these junctions renders lymphatic capillaries highly permeable to interstitial fluids and proteins and allows them to facilitate the migration of immune cells. LECs are bound by anchoring filaments, such as reticular, elastic and collagen fibers, in the extracellular matrix (ECM), allowing for proper lymph flow. These anchoring filaments can stretch to open the lymphatic lumen when the volume of interstitial fluid increases, leading to increased hydrostatic pressure, facilitating the absorption of fluid from surrounding tissue. Lymphatic collector vessels propel lymphatic fluid by the rhythmic contraction of surrounding smooth muscle cells, which are absent around lymphatic capillaries. 

### 1.2. Molecular Regulators of Angiogenesis and Lymphangiogenesis

Both angiogenesis and lymphangiogenesis are stimulated by multiple growth factors, cytokines, chemokines, and inflammatory mediators. Some of them are more specific for one process than the others. The vascular endothelial growth factor (VEGF) family participates in both processes. VEGF-A is the major angiogenic growth factor, mediating its effects by binding to both VEGF-R1 and VEGF-R2 [2]. Similarly, VEGF-C and VEGF-D are the major growth factors which initiate lymphatic vessel formation through their receptor VEGF-R3 [2]. VEGF-C transgenic mice exhibited lymphatic hyperplasia in the skin, suggesting that it has a bigger role in lymphangiogenesis than angiogenesis [3]. Both processes are controlled by a balance of various activating and inhibitory signals. As reviewed by Nyberg et al., there exist a large number of endogenous inhibitors of angiogenesis [4]. They include matrix-derived molecules such as Arrestin and Canstatin (both derived from the non-collagenous domain of collagen type IVα chain), Endorepellin (a Carboxy-terminal derivative of perlecan), Endostatin (a product of Collagen type XV) and Anastellin (a fragment of fibronectin), Fibulin, Thrombospondin (TSP-1 and TSP-2) and Tumstatin; certain growth factors and cytokines such as IFNα, IFNβ, IL-1β, IL-4, IL-12, IL-18, Pigment Epithelium-Derived Factor (PEDF) and Platelet factor (PF)4; and other molecules such as Angiostatin, Vasostatin, Cleaved antithrombin III, Chondromodulin-1 (cartilage derived), 2-Methoxyestradiol (an estradiol metabolite), Plasminogen Kringle 5, Prothrombin Kringle 2, Prolactin (PRL) fragments, Tissue Inhibitors of Metalloproteases (TIMPs), Troponin-1 and s-Flt-1 (the soluble VEGF-R1, that sequesters VEGFs). Finally, both angiogenesis and lymphangiogenesis were shown to be inhibited by the binding of certain semaphorins to their receptors neurophilins and plexins [5]. Semaphorins and plexins are considered as therapeutic targets in cancer [6]. We and others have shown that decorin (DCN), a leucine-rich proteoglycan produced by many mesenchymal cells including chondrocytes and stromal cells in the endometrium and decidual cells in pregnancy, is a major natural inhibitor of angiogenesis [7,8]. DCN is called the “guardian from the matrix” because of its multivalent functions, protecting cells from tumorigenic cues [8].

#### 1.2.1. Angiogenesis: Molecular Regulators

Two VEGF family members, VEGF-A and Placenta Growth Factor (PlGF), are both angiogenic by virtue of binding to their respective high affinity tyrosine kinase receptors (TKRs) VEGF-R2 (also known as KDR) and VEGF-R1 (also known as Flt-1). However, the primary mediators of angiogenesis are VEGF-A and its receptors VEGF-R1 and VEGF-R2, the latter being the primary receptor responsible for the angiogenic action [9]. The process of wound healing is a good example in which both VEGF-A and Fibroblast Growth Factor (FGF)-2 are known to be present at the wound site to stimulate blood vessel development in the affected area [10]. VEGF-A expression during embryonic development is essential; it plays a major role in cardiovascular development and the deletion of VEGF-A is embryonic-lethal [11]. VEGF-A is also important for other physiological processes such as ovulation, pregnancy, and menstruation, and maintenance of blood pressure [1,12]. Hypoxia is a major stimulus for angiogenesis in tissues under physiological and pathological conditions by HIF-1α-mediated induction multiple HIF-1 responsive genes including *VEGF-A* [1]. Other molecules and their respective receptors playing a direct or indirect role in angiogenesis include Platelet Derived Growth Factor (PDGF)/PDGF-R [1], Basic FGF family (bFGF)/bFGF-R [1], Angiopoitins (Ang-1, Ang-2)/Tie-2, ephrin ligands/Ephrin receptors, Tumor Necrosis Factor (TNF)α/TNF-R [1], Transforming Growth Factor (TGF)-β/TGFBR1/II in a context-dependent manner [1,13] and Cardiac Ankyrin Repeat Protein (CARP) [14]. These and other molecular regulators of angiogenesis have been elegantly reviewed by Carmeliet and Jain (2011) [15].

#### 1.2.2. Lymphangiogenesis: Molecular Regulators

It has been shown that among many molecules, VEGF-C is crucial for the development of lymphatic vessels in embryos. In *vegfc*-null mice, endothelial cells can differentiate to form a lymphatic lineage, but this lineage is unable to form mature lymphatic vessels [16]. As a result, these mice die prenatally due to lymphedema. On the other hand, the deliberate overexpression of VEGF-C in transgenic mice leads to lymphatic hyperplasia in the epidermis [3]. The lack of lymphatic vessel sprouting in *vegfc*-null mice could be rescued with the addition of both exogenous VEGF-C and VEGF-D, but not exogenous VEGF-A, suggesting the requirement for the VEGF-C/D receptor VEGF-R3 [16]. In fact, VEGF-R3 is needed for proper lymphatic and cardiovascular development, as *vegfr3*-null mice display lymphatic and cardiovascular defects. VEGF-D, however, has been shown to be expendable for lymphatic development, as *vegfd*-null mice are essentially normal [17]. The absence of VEGF-D is likely compensated for by endogenous VEGF-C, further displaying the importance of VEGF-C in lymphatic development.

Alpha 9/ß1 integrin is another receptor of VEGF-C and VEGF-D, also contributing to lymphangiogenesis [18], and the development of bilateral chylothorax in α*9*/*ß1*-null mice [19]. Neurophilin (NRP) 2 is yet another receptor of VEGF-C and *NRP2*-null mice display abnormal lymphatic systems [20]. Other ligands and receptors that can also directly or indirectly contribute to lymphangiogenesis under a variety of conditions including wound repair, tissue regeneration and tumor development are VEGF-A/VEGF-R2, Fibroblast Growth Factor (FGF)2/FGF-R, Platelet-Derived Growth Factor (PDGF)/PDGF-R, Hepatocyte Growth Factor (HGF)/C-Met, angiopoietin (Ang)1,2/Tie 2, and chemokine and chemokine receptors CCL21/CCR7 and CXCL12/CXCR4 [21,22,23,24,25,26,27,28,29,30]. 

### 1.3. Experimental Models to Investigate Angiogenesis and Lymphangiogenesis

#### 1.3.1. Angiogenesis Models: In Vitro and In Vivo

Functionally, models for both angiogenesis and lymphangiogenesis are very similar, often differing in the molecular markers that are used for identification and quantification. The in vitro models for angiogenesis include the sprouting of endothelial cells from aortic rings placed on fibrin or collagen gel; re-assembly and formation of tube-like structures by various stimulates placed on fibrin gel or Matrigel [31,32]. In vivo models include avascular corneal micro-pockets in rodents that allow the testing of angiogenic compounds, matrigel plugs under the skin and directed in vivo angiogenesis assay (DIVAA) by implanting “angioreactors” under the skin of nude mice [33]. Markers for angiogenesis include VEGF-R1/R2 and CD31 (also known as platelet-endothelial cell adhesion molecule or PECAM-1). Proliferation, migration, and tube formation by VECs (on matrigel or collagen gel) have been used to study molecular regulators angiogenesis in vitro. The most popular VEC is human umbilical vascular endothelial cells (HUVECs) [34], used to study angiogenesis under various conditions, such as breast cancer progression [35]. Moreover, different cancer cell lines have also been used in conjunction with VECs in 2D and 3D (organoid) models in vitro to investigate influence of tumors on angiogenesis and to test drugs on these models [36]. 

A variety of animal models have been used to study angiogenesis in vivo. For instance, zebrafish is a good animal model to study angiogenesis during embryonic development in vertebrates [37,38]. Mouse models are the most popular for studying angiogenesis. Fluorescent reporter transgenic mice have been utilized for in vivo live imaging of angiogenesis and lymphangiogenesis [39]. Methods such as a directed in vivo angiogenesis assay (DIVAA) can quantify angiogenesis in murine models. In this assay, a silicon tube (angioreactor) containing basement membrane extract and experimental angiogenic factor(s) or cells is implanted subcutaneously into the dorsal flank of immunodeficient mice [40]. 

#### 1.3.2. Lymphangiogenesis Models: In Vitro and In Vivo

Markers for lymphangiogenesis typically include VEGF-C, D, and their receptors VEGF-R3 and LEC markers such as lymphatic vessel endothelial hyaluronan receptor 1 (LYVE1), prospero homeobox protein 1(PROX-1) and podoplanin (PDPN). To study lymphangiogenesis, various in vitro assays and models have been developed [41,42]. These include: (a) Freud’s adjuvant-induced lymphatic-rich lesions; (b) explants of thoracic duct fragments allowing LEC sprouting in 3D cultures; (c) LEC differentiation induced in embryoid bodies; and (d) tube formation assays using primary or immortalized human dermal or rat mesenteric LECs [43]. In vivo models of lymphangiogenesis include: (a) wound healing after ear-punches in mice. In this model LEC markers were employed to measure lymphangiogenesis in a spatio-temporal manner [44]; (b) rodent cornea models utilized a surgically created corneal micropocket to stimulate lymphatic ingrowth after injecting a lymphangiogenic test compound together with slow-release polymers [45]. After treatment, this tissue can be immuno-stained for lymphatic endothelial specific markers (LYVE-1, PROX-1, PDPN, VEGF-R3) to identify newly formed lymphatic vessels; (c) we derived a “directed in vivo lymphangiogenesis assay” (DIVLA) [42] from previously established assays for angiogenesis [41]. In this model, small silicon tubes, called angioreactors, are implanted in the dorsal flanks of nude mice and contain cancer cells or lymphangiogenic test molecules immobilized with a basement membrane to initiate the growth of lymphatic vessels into the synthetic tubes. These tubes can then be harvested and lymphangiogenesis can be quantified by measuring mRNAs for specific lymphangiogenic markers, such as LYVE-1, PROX-1 and PDPN, immunostaining for these protein markers and the visual determination of the degree of lymphatic ingrowth identified by the markers. This method can also be used to measure simultaneous angiogenesis by double-labeling for CD31 with lymphatic endothelial markers [42]; (d) and lymphangiogenesis at the tumor site has been quantified by measuring the incidence of lymphatic vessels using immuno histology for LEC makers, as reported for human endometrial carcinomas and lung cancer [46].

### 1.4. Roles of COX-2/PGE2 in Angiogenesis and Lymphangiogenesis

Inflammation is a key mediator of both angiogenesis and lymphangiogenesis. During inflammation, the expression of the enzyme cyclooxygenase (COX)-2 is induced by pro-inflammatory cytokines and other inflammatory mediators. The human COX-2 gene (Ptgs2), located on chromosome 1, is about 8.3 kb long and has 10 exons. Sequence analysis of the 5′-flanking region of the gene has identified several potential transcription regulatory elements, including a peroxisome proliferator response element (PPRE), two cyclic AMP response elements (CRE), a sterol response element (SRE), two nuclear factor kappa B (NF-κB) sites, an SP1 site, a CAAT enhancer binding protein β (C/EBPβ) two AP-2 sites, an E-box, and a TATA box [47]. The transcription factor C/EBPβ was shown to play a critical role in sPLA2IB-induced, receptor-mediated COX-2 gene expression [48]. Lysophosphatidic acid (LPA) is a bioactive phospholipid that is present in all tissues examined to date. LPA signals via cognate G protein-coupled receptors to mediate cellular processes such as survival, proliferation, differentiation, migration, adhesion and vascular development. LPA can promote both vasculogenesis and angiogenesis [49]. Interestingly, LPA also stimulates COX-2 expression and the release of prostaglandins through LPA1, LPA2 and LPA5 by the transcriptional activation of and post-translational stabilization of COX-2 mRNA in ovarian cancer cells. The consensus sites for C/EBP in the COX-2 promoter were essential for the transcriptional activation of COX-2 by LPA [50]. COX-2 activity leads to the production of prostaglandin E2 (PGE2), a downstream signaling molecule mediating a variety of inflammation-associated responses. COX-1 is responsible for constitutive PGE2 production for a variety physiological functions, such as the regulation of blood pressure, gastrointestinal integrity, and fertility. In contrast, the COX-2-mediated production of PGE2 occurs for a short period of time and high local concentration during inflammation that stimulates angiogenesis and lymphangiogenesis for wound healing. These events are recapitulated during tumor progression [51,52]. PGE2 acts on four prostaglandin E2 receptors (EP1–EP4), via both paracrine and autocrine manners. COX-2/PGE2-mediated lymphangiogenesis and angiogenesis are primarily facilitated through the binding of PGE2 to EP2 and EP4. This mediates the production and release of several pro-lymphangiogenic and pro-angiogenic factors such as the VEGF family of growth factors mentioned previously [53], as well as chemokines such as CCL21 [54]. There is a plethora of angiogenic and angiostatic chemokines, some of which are produced at the sites of inflammation [55]. A subgroup of the CXC family chemokines with an ELR-motif in the N-terminal region has the potential to increase angiogenesis via the recruitment of polymorphonuclear neutrophils into inflamed tissue or through the direct stimulation of vascular endothelial cells. CXCR2 is the common receptor, mediating the pro-angiogenic effects of ELR+ chemokines such as CXCL1, CXCL2, CXCL3, CXCL5, CXCL6, and CXCL7. In contrast, Interferon (IFN)-inducible ELR-negative CXC-family chemokines, mostly CXCR3 ligands, such as CXCL4, CXCL9, CXCL10, and CXCL11 are potent angiostatic factors that prevent angiogenesis in response to growth factors and angiogenic chemokines [55].

## 2. Tumor-Associated Angiogenesis and Lymphangiogenesis: Hijacking Inflammatory Mediators

Under physiological conditions COX-1 is constitutively expressed by most cells, allowing steady but slow PGE2 production for many physiological functions, such as maintaining the integrity of the gastric mucosa, mediating normal platelet function, and regulating renal blood flow. In contrast, constitutive COX-2 expression is highly restricted to a few cells in the kidney, reproductive organs and macrophages. COX-2 is the product of an “immediate-early” gene that is rapidly inducible and tightly regulated. During inflammation such as bacterial infection, COX-2 is rapidly induced in all cells at the site of inflammation, which allows local vasodilation and the rapid egress of leukocytes into the tissue spaces to fight the pathogen. Many cancer cells hijack this process, making them highly migratory. COX-2 is overexpressed in most forms of epithelial cancer [56,57,58] leading to high levels of PGE2 production in the tumor microenvironment that facilitate tumor progression and metastasis by multiple mechanisms such as increased tumor cell migration, invasion, tumor-associated angiogenesis, and lymphangiogenesis and the induction of cancer stem cells (CSCs), mostly following the activation of EP2/EP4 receptors [53]. One of the effects of COX-2-mediated EP2/EP4 activation is the upregulation of CCR7, a chemokine receptor that can induce lymphangiogenesis [54]. We found that CCL21/CCR7 expression by breast cancer cells promoted tumor-induced lympho-vascular recruitment in vivo and VEGF-C production by LECs in vitro [59]. The CCL21/CCR7 chemokine axis regulated the expression and secretion of lymphangiogenic factor VEGF-C and thereby promoted proliferation, migration, as well as tube the formation of the primary human LECs. CCR7-mediated VEGF-C secretion by human breast cancer was dependent on protein kinase B (AKT) signaling pathway [59]. LPA, as part of the autotaxin–lysophosphatidic (ATX–LPA) axis, promotes inflammation at tumor sites, allowing for optimal conditions for tumor metastasis [60,61]. Additionally, the ATX-LPA axis allows for the expression of pro-inflammatory cytokines such as IL-8, IL-6, TNF-*α*, VEGF and granulocyte colony-stimulating factor [62]. This study also found that blocking the ATX-LPA in mice bearing breast cancer tumors also decreased the inflammatory responses. LPA was also found to stimulate the expression of COX-2 in myofibroblast cells, which in turn would result in more inflammation at the site of LPA expression [63].

### 2.1. Tumor-Associated Angiogenesis (TAA)

**Figure 2:**
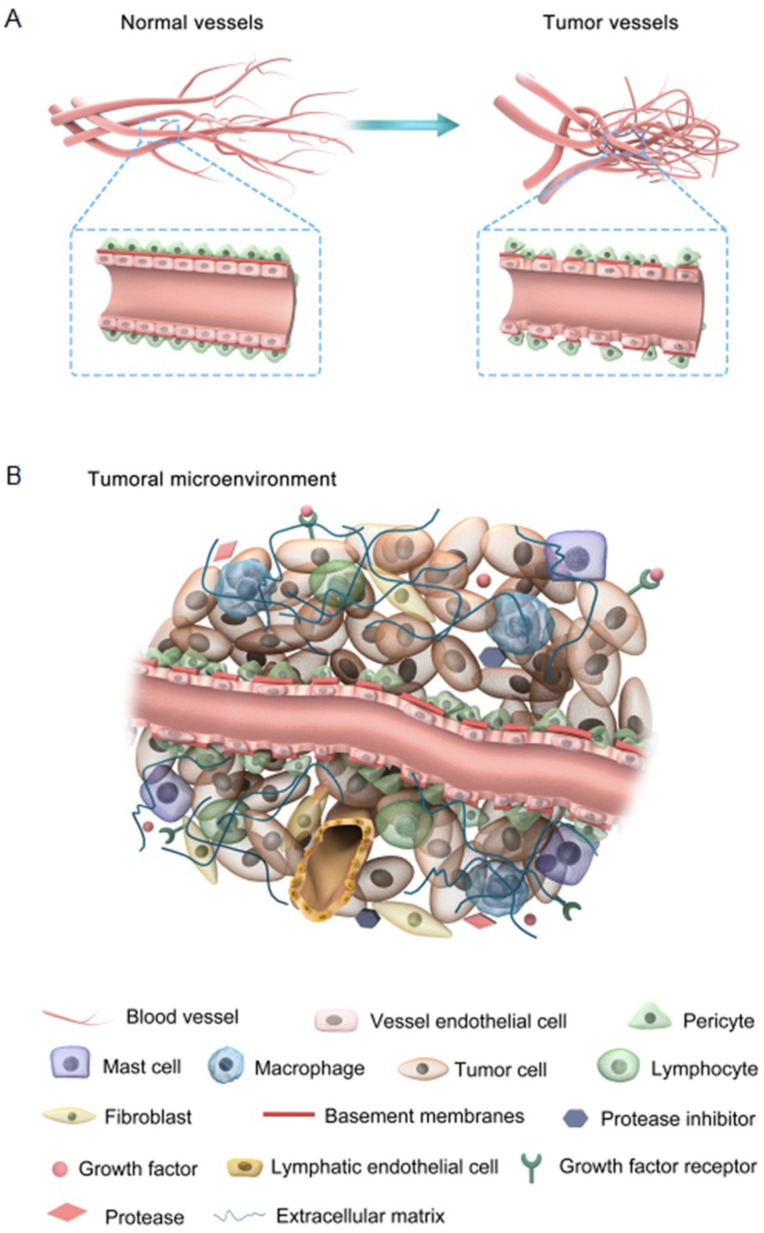
A comparison of normal vs. tumor vasculature (**A**), and constituents of the tumor environment (**B**); tumor-associated angiogenesis is regulated by a plethora of molecules produced by tumor cells, stromal cells and a variety of immune cells in the tumor microenvironment including T cells, NK cells, macrophages and dendritic cells. These molecules include growth factors, cytokines, chemokines, interleukins, lipid derivatives, ECM components and epigenetic regulators such as microRNAs carried by microvesicles [64]. The tumor microenvironment is heterogeneous depending on the tumor type and stage of tumor growth. Figure reproduced with permission from [69].

Angiogenesis is an essential physiological process; however, tumor-associated angiogenesis (TAA) is one of the main hallmarks of cancer progression [60]. In growing tumors, hypoxia is a major mechanism for the upregulation of VEGFs, leading to angiogenesis [64,65]. Cancer can lead to the abnormal development of blood vessels to promote metastasis. Tumor vasculature is often tortuous, hyperpermeable and discontinuous leading to the poor access of systemically administered anticancer drugs [66]. Attempts have been made to “normalize” the tumor vasculature with anti-angiogenic drugs. A plethora of drugs that target vascular endothelial growth factor (VEGF) and its receptor (VEGFR), or other pro-angiogenic pathways, including bFGF, PDGF, Placental Growth Factor (PlGF), Insulin-Like Growth Factor (IGF), Mammalian Target of Rapamycin (mTOR), and histone deacetylases have met with partial success. Direct vascular-targeting with Tumor-Vascular Disrupting Agents (Tumor-VDAs) is distinct from anti-angiogenic strategies and offers a complementary approach. Tumor-VDAs selectively disrupt the immature and rapidly proliferating endothelial cells of established tumor vasculature either by direct apoptotic effects or by effects related to endothelial cell reliance on a tubulin cytoskeleton to maintain cell shape. Tumor-VDAs in preclinical models have shown promise when combined with chemotherapy, radiotherapy, and angiogenesis-inhibiting agents. However, their success in the clinic has been very limited [66]. Anti-angiogenic therapy combined with other modalities has shown some therapeutic benefit [15,67]. Nevertheless, many tumors develop resistance to anti-angiogenic therapies indicating that additional therapeutic targets are needed [68]. Figure 2 compares normal vs. tumor vasculature and constituents of the tumor microenvironment.

As mentioned earlier, PGE2 is a major stimulator of angiogenesis. Inhibiting PGE2 production with a COX1/COX2 inhibitor indomethacin in mice bearing a COX-2 expressing highly metastatic syngeneic mammary adenocarcinoma led to anti-tumor and anti-metastatic effects, in association with a dramatic reduction in TAA in residual tumors [70]. Macrophages at the tumor site are a major source of angiogenic molecules including VEGF. The infiltration of macrophages in breast cancer has been correlated with angiogenesis, increased malignancy and poor prognosis [71,72]. In a polyoma middle T oncoprotein-induced murine breast cancer model [73], macrophage infiltration was shown to be associated with an angiogenic switch. 

### 2.2. Tumor-Associated Lymphangiogenesis (TAL)

During physiological lymphangiogenesis, new lymphatic vessels are formed from pre-existing ones. Similarly, lymphatic networks formed during tumor development utilize pre-existing lymphatics and connect with pre-existing lymph nodes. Like TAA, tumor-associated lymphangiogenesis (TAL) also depends on multiple lymphangiogenic cues such as growth factors, chemokines and cytokines produced by tumor cells and tumor-associated immune cells [74]. These lymphatics allow the drainage of interstitial fluid, as well as the transport of immune cells and cancer cells to the draining lymph node, which forms the nidus for secondary metastasis. Physiological lymphangiogenesis occurs in the post-partum involution of breast tissue in a macrophage-rich stroma with high COX-2 expression, which is believed to drive the progression of ductal carcinoma in situ (DCIS) in post-partum women [75].

**Figure 3:**
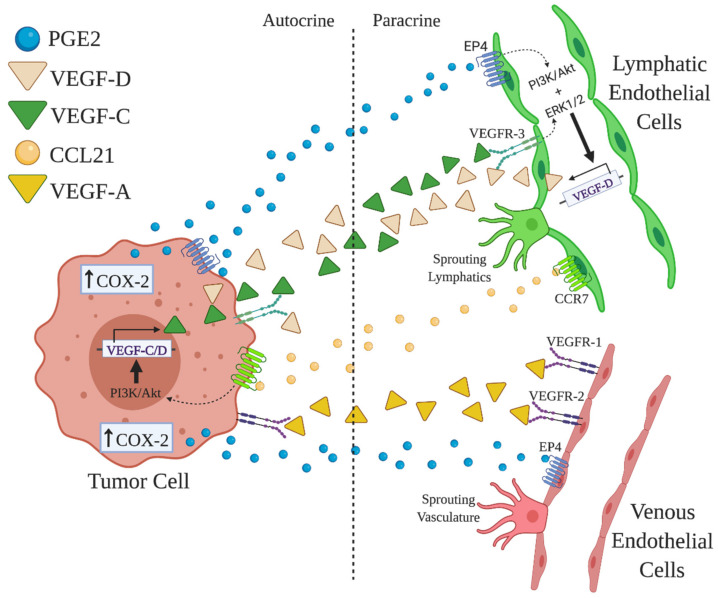
Tumor-associated angiogenesis (TAA) and lymphangiogenesis (TAL) and the proposed roles of PGE2/EP4. vascular endothelial cells (VEC) and LEC precursors located in venules and lymphatic capillaries bear respective receptors VEGF-R1, -R2 for VEGF-A and VEGF-R3 for VEGF-C and -D, respectively. They also bear PGE2 receptor EP4. The production of VEGF family and PGE2 by tumor cells or host cells such as macrophages (not shown) stimulates TAA and TAL by binding to their receptors on VEC or LEC. EP4 activation also induces CCR7 on LEC precursors, which binds to CCL21 produced by tumor or host cells. (Figure based on Tutunea-Fatan et al., 2015 [59] and Nandi et al., 2017 [77]).

Typically, lymphangiogenesis is promoted by VEGF-C released by macrophages [76] and tumor cells [51,72]. In human breast cancer COX-2 expression by both cell classes leading PGE2 production upregulates VEGF-C expression by binding to EP4 receptors [77] (Figure 3). Certain COX-2/EP4-induced microRNAs (miR526b and miR655) can also contribute to the upregulation of VEGFs that induce lymphangiogenesis. The high expression of VEGF-C, VEGF-D and LYVE-1 can be attributed to the overexpression of miR526b and miR655 in human breast cancer [35]. This overexpression in turn enhances the expression of COX-2 and activates EP4 receptor in breast cancer via NFκB pathway [53,78], creating a vicious positive feed-back loop in breast cancer progression. Furthermore, the upregulation of lymphangiogenic factors such as VEGF-C and VEGF-D in lymphoid epithelial cells can promote lymphangiogenesis. A review by Karnezis et al. [79] explains that lymph metastasis is a process of cross-talk between tumor cells and lymphatics through VEGF-C/D and receptors VEGF-R2/3. Iwata et al. [80] reported that the major source of VEGF-C or VEGF-D in mice peritoneum xenografted with human gastric carcinoma were macrophages. Thus, TAL is a result of molecular cross-talk among tumor cells, macrophages and LECs (Figure 3).

### 2.3. Roles of COX-2-Mediated TAA and TAL in Tumor Cell Survival, Nutrition, and Metastasis

The development of new blood and lymphatic vessels is essential for cancer metastasis, as it allows tumors to obtain the nutrients and oxygen required for survival [60,64]. The formation of these vessels allows tumors to grow and disseminate into other parts of the body as metastatic foci. Aggressive forms of pharynx, lung [81], colon [82], breast [57,83,84], and pancreas [85] cancer have all been associated with the overexpression of COX-2 and PGE2 overproduction, which can promote angiogenesis and lymphangiogenesis [52,53]. Certain physiological events, such as childbirth, can have an impact on lymphangiogenesis in breast cancer. For example, a comparison of the degree of lymphangiogenesis in breast tissue in women after birth compared to nulliparous women [75,84] revealed that lymphatic vessel formation was higher in women after giving birth when compared with nulliparous women. Ristimaki et al. also showed that higher levels of COX-2 in breast cancer was associated with cancer hallmarks that result in a poor prognosis and a lower probability of survival [86].

## 3. COX-2-Mediated Molecular Pathways: Key Events in the Synthesis of Prostanoids

**Figure 4:**
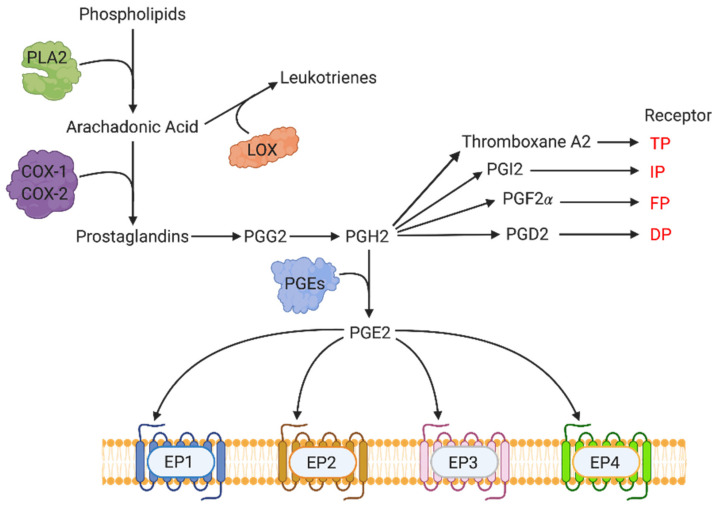
Pathway of prostanoid synthesis and their respective receptors. Phospholipase A2 (PLA2) facilitates arachidonic acid production by acting on membrane phospholipids. Arachidonic acid is converted to many different prostanoids (PGE2, Thromboxane A2, PGI2, PGF2α and PGD2) via the COX-1 and COX-2 enzymes. These prostaglandins then mediate their functions through respective receptors. Figure adapted from Majumder et al., (2018) [53].

COX-2 is a member of the cyclooxygenase family of enzymes, which include COX-1 and COX-3. COX-3 is an isoform of COX-1 that is not present in humans. COX-1 is constitutively expressed in most tissues, while COX-2 is only constitutively expressed in a select set of cell types, mainly of the reproductive and immune system. COX-2 is typically associated with inflammation induced by cytokines, mitogens, and some carcinogens. COX-1 and COX-2 are key enzymes involved in the production of prostanoids (Figure 4). The synthesis of prostanoids begins with the production of arachidonic acid, which is facilitated by phospholipase A2 (PLA2) acting cell membrane phospholipids. Arachidonic acid acts as the substrate for COX-2, and other cyclooxygenases, to produce the prostaglandins PGE2, Thromboxane A2, PGI2, PGF2α, and PGD2. These prostaglandins exert physiological functions by binding to their respective receptors (EP family for PGE2, TP for Thromboxane A2, IP for PGI2, FP for PGF2 α, and DP for PGD2). PGE2 is the most abundant eicosanoid produced downstream of COX by the action of PGE synthase (PGES) enzymes on PGG2. When secreted, PGE2 is a locally active signaling molecule, quickly catabolized by 15-hydroxyprostaglandin dehydrogenase (15-PGDH or HPGD) to the inactive 15-keto-PGE. COX-2 induces TAA and TAL by the downstream production of PGE2 and its binding primarily to EP4 receptor on multiple cells.

The long-chain n-3 polyunsaturated fatty acids (LC n-3 PUFA) of fish oil, eicosapentaenoic (EPA) and docosahexaenoic (DHA) acids are considered cardioprotective owing to their anti-inflammatory actions. Using lipopolysaccharide-stimulated human macrophages, it was shown that EPA and DHA downregulated the production of proinflammatory cytokines associated with the aetiology of metabolic syndrome, NF-κB transcriptional activity and upstream cytoplasmic signalling events [87]. LPA and DHA were also shown to suppress COX-2 activity via the suppression of NF-κB in LPS-treated human umbilical vein endothelial cells [88]. DHA action was further shown to be via the inhibition of NADP(H) oxidase and PKCε [89].

### 3.1. EP Receptors and Molecular Signaling Pathways

**Figure 5:**
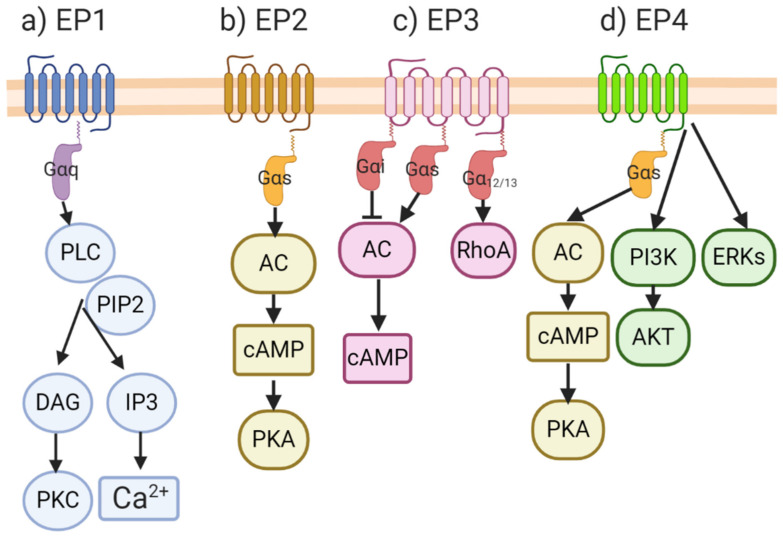
Signaling through the EP family of receptors. EP receptors belong to the family of GPCRs: (**a**) signaling through the EP1 receptor results in the activation of PKC and a higher concentration of intracellular Ca^2+^; (**b**) EP2 receptor signaling induces the production of cAMP and the activation of PKA; (**c**) EP3 is associated with different G proteins: depending on which Gα is associated with EP3, it can increase or decrease cAMP; and activate the Rho family ATPases; (**d**) EP4 and EP2 share the same pathway of PKA activation when PGE2 binds to them. Unlike EP2, signaling through EP4 also stimulates non-canonical activation of the PI3K/AKT and ERK pathways. Figure adapted from Lala et al., (2018) [52].

After PGE2 is produced, it acts on a number of EP receptors (Figure 5), namely EP1, EP2, EP3 and EP4, which are part of a family of membrane bound G-protein coupled receptors (GPCRs) [90,91]. The activation of GPCRs induces the exchange of GTP for GDP, causing the dissociation of the complex into alpha (α) and beta/gamma (β/γ) subunits, and the activation of downstream second messengers. Each EP receptor binding event results in the production of different downstream molecules that have different physiological and molecular consequences. The activation of the EP1, which is coupled with Gαq, results in the cleavage of PIP2 by phospholipase C (PLC) action. This results in the production of the second messengers IP3 and DAG, which leads to higher cytosolic calcium via the opening of calcium-gated channels in the endoplasmic reticulum. EP3 is associated with different G proteins: Gαi, which is responsible for adenylyl cyclase (AC)/cAMP inhibition; Gαs, which is responsible for stimulating cAMP production; or Gα12/13, which stimulates Rho family GTPases. Both the EP2 and EP4 receptors are associated with Gαs, and upon release, activate AC, resulting in cAMP production that culminates in protein kinase A (PKA) activation. EP4 activation, unlike EP2, also results in the non-canonical stimulation of the PI3K/Akt and ERK pathways. A large number of EP receptor agonists and antagonists with selective binding abilities for the individual EP receptors and their pharmacokinetic properties, have been reported in the literature [92], a listing which is beyond the scope of the present review. 

### 3.2. COX-2/EP Receptors and Breast Cancer

The disruption of PGE2 homeostasis, by overactive COX-2, for example, is associated with many physiological conditions such as chronic inflammation, Alzheimer’s disease, and tumorigenesis. As previously discussed, COX-2 is a commonly overexpressed in many aggressive forms of cancer [81,82,83,85,93]. The causal relationship of COX-2 to tumor progression has been demonstrated by ectopic overexpression [94] and the knockdown [95] of the *COX-2* gene and use of COX-2 inhibitors [96]. A large number of studies show that COX-2 promotes tumor initiation, progression and metastasis of most epithelial cancers [96]. Furthermore, selective and even non-selective inhibitors of COX-2 showed protective effects against colorectal and mammary carcinogenesis [96,97,98,99,100,101]. In breast cancer, increased COX-2 expression signals poor prognosis, and is associated with high tumor cell proliferation rates, high histological grade, ductal carcinoma in situ, elevated p53 expression, HER-2 amplification, and axillary node involvement [86]. COX-2 expressing murine mammary tumor transplants [102,103] and spontaneous mammary tumors in female C3H/HeJ mice [104] when treated with drugs inhibiting both COX-1/COX-2, resulted in reduced tumor growth as well as metastasis.

Many studies have shown that the overexpression of COX-2 leads to elevated endogenous PGE2, promoting breast cancer progression through multiple mechanisms: inactivation of host anti-tumor immune cells [102,103,104,105], enhanced cancer cell migration [70,106], invasiveness [70,107], tumor-associated angiogenesis [70,108], via multiple angiogenic pathways, and tumor-associated lymphangiogenesis [51,72,77,109] through the upregulation of VEGF-C and VEGF-D. In many studies conducted in our laboratory, the primary mediator of these events can be attributed to the activation of the PGE2 receptor EP4 on tumor and various host cell classes.

EP4 activity promotes breast cancer cell migration [106], invasion [107], angiogenesis and lymphangiogenesis at the tumor site [51,72,77,109]. We also observed that in COX-2 expressing breast cancer cells, inducible nitric-oxide synthase (iNOS) was upregulated through PGE2/EP4 activity in a cGMP/PKG-dependent manner [107]. Estrogen and progesterone receptor-positive breast cancer patients with elevated EP4 expression were more likely to be non-responsive to neoadjuvant endocrine therapy [110]. This study also showed that epigenetic alteration leading to the overexpression of EP4 was crucial for estrogen-independent growth, through the activation of the ERα-cofactor CARM1. 

Other studies revealed that the activation of EP4 receptors on host immune cells and endothelial promoted tumor progression. For example, EP4 activity in natural killer (NK) cells [105,111,112] and T cells [113] blocked their killer functions. In macrophages, EP4 promoted immune suppressor functions [114], and in dendritic cells, EP2/EP4 activity blocked their antigen presenting functions. Furthermore, EP4 activity in tumor-associated macrophages increased lymphangiogenic activity by the upregulation of VEGF-C or VEGF-D [72]. Similarly, PGE2-mediated EP4 activation in host LECs stimulated LEC proliferation, migration, and tube formation, triggered by upregulation of VEGF-C or VEGF-D and VEGFR3, culminating in the promotion of lymphangiogenesis [77].

Lastly, we found that COX-2/EP4 activity also induced and sustained stem-like cells (SLCs) in breast cancer cells, shown in a both syngeneic murine breast cancer model and human breast cancer cells [72]. Findings from other studies support these results in a different murine breast cancer model [115]. SLCs are a minor subpopulation of tumoral cells, characterized by an unlimited capacity of self-renewal [116,117], and resistance to conventional chemo/radiotherapies, leading to the recurrence of both primary and metastatic tumors [118,119]. These studies reveal the plastic phenotype of these SLCs, with their tumorigenic functions regulated by the microenvironment and that EP4 is a good therapeutic target for SLC ablation or suppression.

While COX-2 is an excellent therapeutic target for treating breast cancer in combination with other drugs, thrombo-embolic side effects of COX-2 inhibitors noted after prolonged use in arthritis patients [120,121] calls for alternative targets downstream of COX-2 that may spare the side effects. The following reasons suggest that EP4 is a suitable target: (i) as listed above, EP4 expressed by cancer cells and multiple host cells plays a key role in COX-2-mediated breast cancer progression; (ii) many physiological functions of EP4 shared by EP2 via PKA stimulation [91,122] suggest the relative redundancy of EP4; (iii) non-conical signaling by EP4, not shared by EP2, involves PI3k/Akt pathway promoting cancer cell survival including the survival of stem-like cells, induced by EP4 activity (Figure 5d); (iv) targeting EP4 spares EP3-mediated vasoprotection by PGE2 and PGI2 receptor (IP)-mediated vasoprotection by PGI2, as suggested by findings in a variety of animal models of cardiac ischemia. For example, using IP-null and Thrombospondin receptor (TP)-null mice, it was shown that IP but not TP receptor was cardio protective. Endogenous PGI2, produced during cardiac ischemia/reperfusion, mediated a protective effect on cardiomyocytes independent of its effects on platelets and neutrophils [123]. Moreover, PGE2 was shown to mediate cardio protective effects via EP3 receptor activation. Ischemic myocardial injury could be reduced in transgenic mice with the cardio-specific overexpression of the EP3 receptor [124]. Similarly, structurally diverse EP3 agonists could reduce myocardial infarct size in rats. This amelioration was mediated by PKC activation and the opening of KATP (ATP-sensitive K) channels [125]. 

Triple-negative breast cancer (TNBC), accounting for about 15% of human breast cancers, is known to be highly aggressive and resistant to conventional therapies due to their lack of well-known targets [126]. We found that TNBCs are mostly COX-2-positive [53], also reported by other investigators [127]. TNBC represents the deadliest form of breast cancer, which resists cytotoxic therapies. Gene expression patterns that were gleaned from publicly available databases suggested that TNBCs expressed multiple drug resistance-associated protein (MRP) 4 (an active PGE2 exporter), low PGT (a PGE2 importer), and low 15-PGDH (a PGE2 catabolizer) [128]. They collectively favored the maintenance of high levels of PGE in the tumor microenvironment that may contribute to poor therapeutic response, indicating that PGE2 inhibitors may potentiate therapeutic response. We suggest that EP4 antagonists should improve the therapeutic response of TNBC to other therapeutic modalities.

### 3.3. Roles of COX-2/EP4 in TAA and TAL

We showed that the overexpression of COX-2 mediates the production of PGE2, a prostaglandin that binds to and activates prostaglandin E receptors (EP1–EP4). It has also been shown that high COX-2 levels in post-partum breast cancer lead to higher incidences of tumors associated with lymphangiogenesis [84]. Multiple studies have uncovered the promoting roles of COX-2 on TAA and TAL. For instance, in BALB/c nu/nu mice bearing a gastric carcinoma cell line, treatment with a COX-2 antagonist resulted in reduced levels of the lymphangiogenic marker LYVE-1, and the angiogenic marker PECAM-1 within the gastric walls of the mice [80]. The overexpression of COX-2 promoted angiogenesis in colorectal cancer by an increased production of VEGFs and basic fibroblast growth factor (bFGF) [58]. COX-1/COX-2 knockout murine models revealed that host-derived COX-2 but not COX-1 promoted tumor growth, metastasis and VEGF production [129]. In addition, our studies with COX-2 and HER-2 expressing human breast cancer specimens and cell lines revealed that COX-2 rather than HER-2 was responsible for VEGF-C upregulation and TAL [130]. These studies establish the important role of COX-2 in promoting angiogenesis and lymphangiogenesis, regardless of cancer phenotype. As reviewed in Section 2.1 and Section 2.2, the promoting roles of COX-2 in both TAA and TAL are primarily mediated by PGE receptor EP4. EP4 activation can trigger other pathways both for angiogenesis and lymphangiogenesis. Using in vitro studies of lymphangiogenesis with a rat mesenteric LEC cell line and in vivo studies of PGE2-induced lymphangiogenesis in angioreactors placed under the skin of nude mice, we demonstrated the roles of tumor as well as host-derived PGE2 in inducing lymphangiogenesis, at least in part, by activating EP4 and VEGFR-3 on LECs [77]. EP4, being a common target on both tumor and host cells contributing to tumor-associated lymphangiogenesis, reaffirms the therapeutic value of EP4 antagonists in the intervention of lymphatic metastasis in breast cancer.

### 3.4. Roles of COX-2/EP4-Induced microRNAs (miRNA)s in TAA and TAL

COX-2 overexpression in tumors has been linked to the expression of certain oncogenic miRNAs, two of which are miR526b and miR655. Both miR526b and miR655 were shown to be highly upregulated in COX-2 overexpressing MCF7-COX2 breast cancer cell line, which was ER, PR and HER2-negative [78,131]. These two miRNAs were shown to induce SLC-phenotypes in breast cancer cells, as well as stimulate angiogenesis and lymphangiogenesis [53,78]. Later, Hunter et al. [35] tested the angiogenic and lymphangiogenic effects of these two miRNAs on the poorly metastatic, ER expressing MCF-7 breast cancer cell line by the ectopic overexpression of the miRNAs. miRNA overexpressing cells exhibited an increased expression of angiogenic and lymphangiogenic markers, VEGFs and EP4 receptors. miRNA-high cells produced angiogenic factors, as demonstrated by the promotion of migration and tube formation by HUVECs treated with conditioned media from both miRNA overexpressing cell lines. Although the exact mechanism by which miR526b and miR655 stimulate angiogenesis is not fully understood, it was shown that the expression of these two miRNAs causes COX-2 overexpression through the NF-κB pathway, which in turn results in a higher expression of these miRNAs, promoting a positive feedback loop between both miRNAs and COX-2 [35,53,78]. 

MicroRNAs inhibit the expression of their target genes by inhibiting their transcription or promoting mRNA degradation. We found that the single common target of miR526b and mir655 is “Cytoplasmic Polyadenylation Element-Binding Protein 2 (CPEB2)”. CPEB2 was shown to have strong tumor suppressor properties [132]. siRNA-mediated CPEB2 knock-down in MCF-7 mammary carcinoma cells or CRISPR-Cas9-mediated CPEB knock-out in non-tumorigenic mammary epithelial cell line MCF10A led to increased migration, proliferation, invasion and stem cell properties. CPEB2 knocked-out MCF10A cells became tumorigenic and metastatic in NOD/SCID/IL2Rϒ-null mice. While there was no scope for evaluating anti-angiogenic functions of CPEB2 in this study, the tumor suppressor properties of CPEB2 were ascribed to its splice variant CPEB2A. CPEB interacts with elongation factor 2 (eEEF2) to impede HIF-1α translation [133]. HIF1-α is an angiogenesis promoting transcription factor, thus it is highly likely that CPEB2 is anti-angiogenic. PTEN is a tumor suppressor gene that inhibits the action of the HIF1-α [134,135], which, when expressed, can lead to the expression of angiogenic factors, such as VEGF [136]. In both miR526b and miR655 expressing MCF-7 cell lines, the expression of PTEN is reduced [35] and both miRNA regulate the expression of HIF1-α in breast cancer [57]. In a more recent publication, the expression of HIF1-α was compared among different breast cancer cell lines that expressed miR526b or miR655 [65]. The higher production of this angiogenic transcription factor was observed in cells that expressed these miRNAs when they were put under hypoxic conditions induced by cobalt chloride. Furthermore, the expression of VHL, which downregulates HIF1-α, was lower in cell lines expressing miR655 and miR526b. The treatment of miRNA-overexpressed cells with COX-2/EP4 and PI3K/AKT inhibitors resulted in inhibition of the hypoxia-induced phenotypes previously seen in these cells. This further establishes the role of COX-2/EP4 in the expression and functions of these oncogenic and angiogenesis-promoting miRNAs.

## 4. COX-2/EP4 as Therapeutic Targets in Breast Cancer: Experimental Models

### 4.1. Spontaneous and Syngeneic Murine Breast Cancer Models

#### 4.1.1. Spontaneous Murine Breast Cancer

Spontaneous murine breast cancer models have been used earlier to explore the roles of COX in tumor progression and TAL. In spontaneous mammary tumors arising in retired breeder C3H/HeJ mice, we observed that both COX-1 and COX-2 promoted tumor progression and metastasis and tumor-associated angiogenesis. Chronic oral administration of a non-selective COX1/COX2 inhibitor indomethacin in retired breeder female mice delayed spontaneous mammary tumor development, reduced spontaneous lung metastasis from the primary site and prolonged animal survival. Residual tumors in treated mice exhibited high lymphocyte infiltration and scanty angiogenesis [104]. HER2/neu transgenic mice exhibit increased spontaneous mammary tumors, which are highly vascularized. However, HER2/neu-induced tumorigenesis and angiogenesis are drastically reduced in COX-2 knockout mice [137].

#### 4.1.2. Syngeneic Murine Breast Cancer Models Using Cell Lines

We derived metastatic variant clones from C3H/HEJ spontaneous tumors. A highly metastatic variant produced from a clone C3 by five cycles of in vivo passage of tumor cells from the subcutaneous site to the lungs, named C3L5 expressed high levels of COX-2. Phenotypically this cell line is equivalent to human triple-negative breast cancer (TNBC). We found that COX inhibitors retarded the growth and metastasis of C3L5 tumor cells transplanted at the mammary site by the inhibition of cancer cell migration, invasiveness and angiogenesis [70]. We found that like human TNBC lines, EP2 activity shared by EP4 promoted the migration of this cell line [106]. We further observed that the PGE2-mediated invasiveness of this cell line was mediated by EP4 activation associated with the upregulation of iNOS [107]. The C3L5 cell line transplanted at the mammary site caused spontaneous metastasis to the lung and lymph nodes by day 12 of transplantation in syngeneic C3H/He J mice [51]. Both selective COX-2 inhibitor celecoxib and a selective EP4 antagonist, but not an EP1 antagonist, were highly effective in abrogating tumor growth, angiogenesis, lymphangiogenesis and metastasis to lung and lymph nodes in vivo [51]. Subsequently Majumder et al. [72] made use of this syngeneic breast cancer model to assess angiogenesis and lymphangiogenesis driven by VEGF-C/D produced by tumor-associated macrophages (TAMs) in vivo and the induction of stem-like cells (SLC)s in vitro. Results revealed that EP4 is an excellent therapeutic target to block stem-like properties in cancer cells and tumor-associated angiogenesis and lymphangiogenesis induced by VEGF-A/C/D production by cancer cells as well as TAMs. Using a metastatic variant of the syngeneic mammary tumor cell line 410 in BALB/C mice that expresses both COX isoforms, Kundu et al. (2002) showed that the oral administration of either a selective COX-2 or COX-1 inhibitor to mice with established tumors resulted in the significant inhibition of tumor growth and metastasis [138]. Subsequently, they demonstrated that treating the cells with EP4 antagonist in vitro drastically abrogated their tumorigenesis in the lungs following intravenous inoculation [139]. PGE2 was shown to suppress NK cell function in this model by EP4 activation [111]. An EP4 antagonist could abrogate PGE2-mediated immunosuppression of NK cells and inhibit the metastasis of cancer cells in this model [112]. They further demonstrated in this model that EP4 is a therapeutic target for stem-like cells [115]. Another mechanism of PGE2/EP4 meditated tumor progression is induction of myeloid-derived suppressor cells (MDSCs) of the non-macrophage lineage (polymorphonuclear and mononuclear leukocytes). Albu et al. (2017) demonstrated an increased number of MDSCs in CT-26 colon cancer–bearing mouse spleen and reported that the chronic treatment of the cancer-bearing mice with an EP4 antagonist resulted in the abrogation of this rise, associated with strong anti-tumor effects [140].

### 4.2. Human Breast Cancer Cell Line and Patient-Derived Xenografts in Immune-Deficient Mice 

Many studies have utilized human breast cancer line xenografts in immune-deficient mice to test the therapeutic effects of drugs. Nude mice are deficient in T and B cells but contain functional NK cells. We utilized the nude mouse model for the successful immunotherapy of human melanoma xenografts with non-selective COX inhibitor indomethacin given orally in combination with systemic IL2 [141]. Many investigators have used the nude mouse model to investigate the role of COX-2 in breast cancer metastasis. For example, Singh et al. (2007) overexpressed COX-2 in human breast cancer cell line MDA-435S to show that the cells exhibited enhanced the bone metastasis by high PGE production and a COX-2 inhibitor, MF-tricyclic, inhibited bone metastasis caused by a bone-seeking clone [142]. NOD/SCID/IL2Rϒ- null (NSG) mice which lack in functional T, B and NK cells have also been used for grafting human tumor cell line xenografts for therapeutic purposes. We used these mice to test the stem cell induction in COX-2 overexpressing human MCF-7 breast cancer cell lines (MCF-7-COX2 cells) tested in vivo by implanting limiting cell numbers at the mammary site over successive transplant generations. These cells are ER-/PR-/Her-2- (TNBC phenotype) and highly EP4 expressing. Treating the cells in vitro with an EP4 antagonist or knocking down *EP4* abrogated their lung colonizing ability and tumorigenicity after intravenous inoculation [143]. 

**Figure 6:**
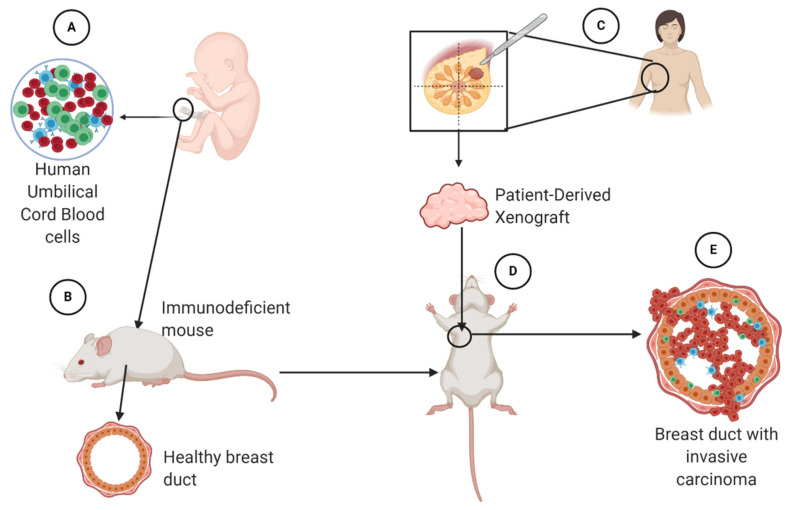
Designing a humanized mouse model using umbilical cord blood cells (**A**). These cells are introduced into a healthy immunodeficient mouse (**B**), so that it will develop human immune cells. Human xenografts from triple-negative breast cancer (TNBC) patients can be obtained (**C**) and transplanted (**D**) into the mouse model at the mammary site. The goal is for the mouse to develop TNBC (**E**) so that it is possible to observe an interaction between human cancer cells and human lymphocytes in this model.

Despite their usefulness in research, translating results from these mouse models to the clinic can be difficult due to the differences between human and murine immune systems [144]. In attempts to overcome this issue, tumors obtained from human patients have been successfully xenografted into immunodeficient NSG mice “humanized” by transplanting human umbilical cord-derived CD34 + hematopoietic stem cells [145,146]. The xenografts faithfully recapitulate the phenotype and genotype of the parental tumors. These mice produce human immune cells allowing interaction between a human tumor and human immune cells at the tumor site (Figure 6). An advantage of this tool is that researchers can follow a variety of tumors with different molecular profiles from different patients in a human-like model. These models can be self-sustainable and used, in theory, for as long as necessary [147]

### 4.3. Patient-Derived Xenografts of Triple-Negative Breast Cancer (TNBC)

Triple-negative breast cancer remains one of the hardest forms of breast cancer to treat, resisting cytotoxic and radiation therapies. Patient-derived TNBC xenografts, or TNBC-PDXs, represent a powerful tool that has allowed scientists to test newer drugs for aggressive cancers such as TNBCs. Jackson Laboratories maintain humanized NSG (Onco-HuNSG) mice and a good repository of human TNBC-PDX in these mice. Wang et al. [148] have shown the usefulness of anti-PD-1 therapy in TNBC-PDX model. 

### 4.4. EP4 Antagonists Used in Combination Therapies

**Figure 7:**
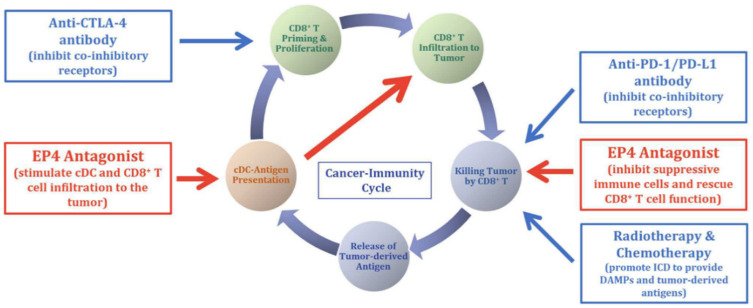
Schema for the rationale of using EP4 antagonists in combination with other therapies, including immune check point inhibitors such as anti-CTLA-4, anti-PD1/PD-L1 in cancer therapy. Red boxes indicate the roles EP4 on tumor-associated T cells. (Reproduced with kind permission from Take et al., 2020) [154].

We suggest that EP4 antagonists will be most effective when used in combination with other therapies (Figure 7), in particular with immune checkpoint inhibitors (ICIs). Programmed cell death (PD)-1 is a checkpoint protein on T lymphocytes, serving as physiological “off switch” preventing them from attacking other cells in the body, which produce its ligand PD-L1. Some cancer cells hijack this protection from T cells by producing large amounts of PD-L1, even if the T cells can recognize tumor-associated antigen. This defense mechanism is exploited by many solid tumors, leading to a revived interest in immunotherapy with immune checkpoint (PD-1, PD-L1, CTLA-4) inhibitors (ICIs). Anti-CTLA4 and anti-PD-1 therapies have resulted in tumor regression and prolonged animal survival in murine colon and breast cancer models [149]. PD-L1 expression in breast cancer including TNBCs is heterogeneous and usually associated with an abundance of tumor-infiltrating lymphocytes. Recent clinical trials in breast cancer with PD-1/PD-L1 inhibitors have shown promising results on tumor responses and/or disease control, notably in the triple-negative subtype [150,151,152]. 

Based on our success with EP4 antagonists applied in syngeneic murine TNBC models [51,72] and human TNBC lines in NSG mice [143], we tested the therapeutic efficacy of an EP4 antagonist (AAT-008, courtesy of Ask/AAT Japan, currently being produced by Arrys therapeutics, USA) in TNBC-PDX in humanized NSG mice from Jackson Labs. Our preliminary data revealed significant anti-tumor effects (70% reduction in mean tumor volume on day 40 of treatment) with this EP4 antagonist, administered by oral gavage (15 mg/kg, once a day) in established TNBC–PDX derived from a single patient (*n* = 4, mice in each group, control vehicle treated vs. drug-treated mice) [Lala PK and Koropatnick J, unpublished data]. We are currently testing TNBC-PDXs from multiple patients and exploring the immune cell phenotype in the tumors in vehicle-treated vs. drug-treated mice, and plan to test EP4 antagonist in combination with PD-1 and PD-L1inhibitors.

Given the multiple tumor-promoting roles of EP4 expression on tumor cells and host immune cells and endothelial cells, we suggest that the combination of EP4 antagonists and other immunomodulating drugs should work synergistically, especially in TNBCs displaying a diverse microenvironment. Excellent reviews by Ching et al. [153] and Take et al. [154] strongly suggest the potential benefit of EP4 antagonist in combination with other immunomodulating agents in cancer therapy. We suggest that EP4 antagonists should enhance the efficacy of ICIs since their mechanisms of action are complementary as listed earlier. While ICIs act by T cell activation, EP4As also activate NK, macrophage and dendritic cells, turning an “immune-cold” tumor microenvironment into “immune-hot” to potentiate the effects of ICIs. This is an unmet need of existing immunotherapy regimens (Figure 7).

### 4.5. Currently Ongoing Human Trials with EP4 Antagonist as Single Agent or Combination Therapies

EP4 antagonist AAT-007 (produced by Ask/At pharma, Nagoya, Japan) was used in phase 1 and 2 human trials in more than 800 arthritis patients in USA, which was well tolerated in pharmacologically effective doses (300 mg orally twice daily), with no evidence of dose-limiting toxicity (Dr. Yukinori Take, Ask/At, Japan, personal communication, cited with permission). Since then, another more potent EP4 antagonist, named AAT-008, was reported by Okumura et al. [155] at Ask/At pharma in Japan. This compound has not yet been tested on humans but has a better bioavailability than AAT-007. Currently, there are multiple clinical trials using other EP4 antagonists. In a recent multi-center human study in France and the USA, an EP4 antagonist E7046 (Trial registration number NCT02540291) administered orally once daily, demonstrated manageable tolerability, immuno-modulatory effects, and a best response of stable disease (≥18 weeks) in several patients being treated for advanced malignancies [156]. TPST-1495 is being used to treat TNBC, colorectal cancer, lung adenocarcinoma, head and neck squamous cell carcinoma, bladder cancer, and gastric cancer. This trial is still recruiting patients and is set to begin on August 2021 (Trial identifier: NCT04344795, clinicaltrials.gov). Other clinical trials using several EP4 antagonists along with other therapeutic modalities in a variety of cancers include: Clinical Trials ID: NCT03658772, NCT03696212, NCT03152370, NCT03661632).

## 5. Conclusions and Future Directions

**Figure 8:**
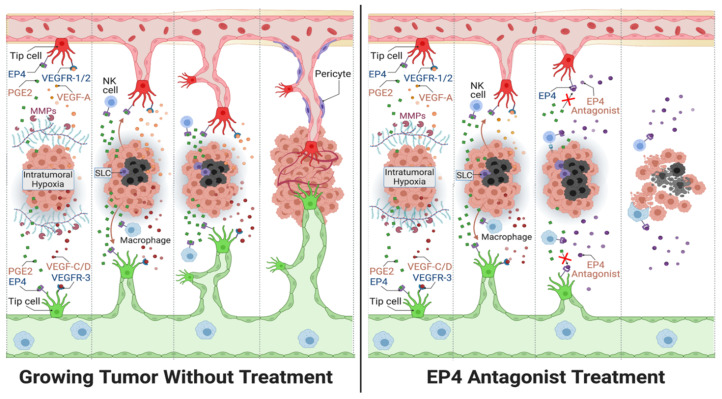
General outline for the action of EP4 antagonists used in breast cancer treatment (**right** panel) compared to conditions of no treatment (**left** panel). The diagram includes the interaction of EP4 agonist PGE2 with EP4 receptors on sprouting endothelial cells (tip cells), natural killer (NK) cells and macrophages, along with VEGFs binding to VEGF receptors (VEGFRs) located on the tip cells of newly formed lymphatics (green) and blood vessels (red). VEGFs are released by hypoxia induction within the tumor core (black cells). Also, the presence of matrix metalloproteases (MMPs) enhances tip cell invasion into the extracellular matrix to complete vasculature. The right panel demonstrates how EP4-antagonist treatment abrogates both angiogenesis and lymphangiogenesis. Furthermore, EP4 antagonist treatment prevents the stimulation of cancer stem-like cells (SLCs) in breast cancer. Lastly, antagonizing EP4 receptors located on various immune cells (NK cell and macrophages) allow an enhanced anti-tumor immune response.

Angiogenesis and lymphangiogenesis are only a part of the tumor microenvironment. The initial expectations of treating vascular tumors with traditional angiogenesis pathway inhibitors as single agents have not been fulfilled, calling for the search of novel therapy targets for use in combination therapy [68]. Drugs that can block multiple tumor-associated events, including angiogenesis and lymphangiogenesis, hold better potential for success. EP4 antagonist can significantly block tumor-associated angiogenesis and lymphangiogenesis in breast cancer (Figure 8). EP4 antagonists present as promising drugs in combination with other immuno-stimulatory agents such as ICIs. Molecular profiling including genomics and immunophenotyping could be used to personalize treatments for patients with TNBCs, which are known to be highly diverse in genotype. In addition, potential tumor markers for aggressive forms of cancer may help early diagnosis and therapeutic monitoring. As discussed in this review, COX-2-induced miRNAs miR655 and miR526b enhances tumor-associated angiogenesis and lymphangiogenesis [35,65] and can serve as biomarkers for breast cancer. In conclusion, the evidence highlighted in this review provides a rationale for the pursuit of EP4 antagonists in combination therapies to treat TNBC and other cancers that display high levels of angiogenesis and lymphangiogenesis.

## Data Availability

Not applicable.

## References

[1] Otrock Z., Mahfouz R., Makarem J., Shamseddine A. (2007). Understanding the Biology of Angiogenesis: Review of the Most Important Molecular Mechanisms. Blood Cells Mol. Dis..

[2] Shibuya M. (2011). Vascular Endothelial Growth Factor (VEGF) and Its Receptor (VEGFR) Signaling in Angiogenesis: A Crucial Target for Anti- and Pro-Angiogenic Therapies. Genes Cancer.

[3] Jeltsch M. (1997). Hyperplasia of Lymphatic Vessels in VEGF-C Transgenic Mice. Science.

[4] Nyberg P., Xie L., Kalluri R. (2005). Endogenous Inhibitors of Angiogenesis. Cancer Res.

[5] Neufeld G., Kessler O. (2008). The Semaphorins: Versatile Regulators of Tumour Progression and Tumour Angiogenesis. Nat. Rev. Cancer.

[6] Worzfeld T., Offermanns S. (2014). Semaphorins and Plexins as Therapeutic Targets. Nat. Rev. Drug Discov..

[7] Lala P.K., Nandi P. (2016). Mechanisms of Trophoblast Migration, Endometrial Angiogenesis in Preeclampsia: The Role of Decorin. Cell Adhes. Migr..

[8] Neill T., Schaefer L., Iozzo R.V. (2012). Decorin. Am. J. Pathol..

[9] Ferrara N., Gerber H.-P., LeCouter J. (2003). The Biology of VEGF and Its Receptors. Nat. Med..

[10] Nissen N.N., Polverini P.J., Koch A.E., Volin M.V., Gamelli R.L., DiPietro L.A. (1998). Vascular Endothelial Growth Factor Mediates Angiogenic Activity during the Proliferative Phase of Wound Healing. Am. J. Pathol..

[11] Carmeliet P., Ferreira V., Breier G., Pollefeyt S., Kieckens L., Gertsenstein M., Fahrig M., Vandenhoeck A., Harpal K., Eberhardt C. (1996). Abnormal Blood Vessel Development and Lethality in Embryos Lacking a Single VEGF Allele. Nature.

[12] Brown L.F., Yeo K.T., Berse B., Yeo T.K., Senger D.R., Dvorak H.F., van de Water L. (1992). Expression of Vascular Permeability Factor (Vascular Endothelial Growth Factor) by Epidermal Keratinocytes during Wound Healing. J. Exp. Med..

[13] Pardali E., Goumans M.-J., ten Dijke P. (2010). Signaling by Members of the TGF-β Family in Vascular Morphogenesis and Disease. Trends Cell Biol..

[14] Shi Y., Reitmaier B., Regenbogen J., Slowey R.M., Opalenik S.R., Wolf E., Goppelt A., Davidson J.M. (2005). CARP, a Cardiac Ankyrin Repeat Protein, Is Up-Regulated during Wound Healing and Induces Angiogenesis in Experimental Granulation Tissue. Am. J. Pathol..

[15] Carmeliet P., Jain R.K. (2011). Molecular Mechanisms and Clinical Applications of Angiogenesis. Nature.

[16] Karkkainen M.J., Haiko P., Sainio K., Partanen J., Taipale J., Petrova T.V., Jeltsch M., Jackson D.G., Talikka M., Rauvala H. (2004). Vascular Endothelial Growth Factor C Is Required for Sprouting of the First Lymphatic Vessels from Embryonic Veins. Nat. Immunol..

[17] Baldwin M.E., Halford M.M., Roufail S., Williams R.A., Hibbs M.L., Grail D., Kubo H., Stacker S.A., Achen M.G. (2005). Vascular Endothelial Growth Factor D Is Dispensable for Development of the Lymphatic System. MCB.

[18] Vlahakis N.E., Young B.A., Atakilit A., Sheppard D. (2005). The Lymphangiogenic Vascular Endothelial Growth Factors VEGF-C and -D Are Ligands for the Integrin A9β1. J. Biol. Chem..

[19] Huang X.Z., Wu J.F., Ferrando R., Lee J.H., Wang Y.L., Farese R.V., Sheppard D. (2000). Fatal Bilateral Chylothorax in Mice Lacking the Integrin A9β1. Mol. Cell. Biol..

[20] Yuan L., Moyon D., Pardanaud L., Bréant C., Karkkainen M.J., Alitalo K., Eichmann A. (2002). Abnormal Lymphatic Vessel Development in Neuropilin 2 Mutant Mice. Development.

[21] Karpanen T., Egeblad M., Karkkainen M.J., Kubo H., Ylä-Herttuala S., Jäättelä M., Alitalo K. (2001). Vascular Endothelial Growth Factor C Promotes Tumor Lymphangiogenesis and Intralymphatic Tumor Growth. Cancer Res..

[22] Kubo H., Cao R., Brakenhielm E., Makinen T., Cao Y., Alitalo K. (2002). Blockade of Vascular Endothelial Growth Factor Receptor-3 Signaling Inhibits Fibroblast Growth Factor-2-Induced Lymphangiogenesis in Mouse Cornea. Proc. Natl. Acad. Sci. USA.

[23] Chang L.K., Garcia-Cardena G., Farnebo F., Fannon M., Chen E.J., Butterfield C., Moses M.A., Mulligan R.C., Folkman J., Kaipainen A. (2004). Dose-Dependent Response of FGF-2 for Lymphangiogenesis. Proc. Natl. Acad. Sci. USA.

[24] Cao R., Björndahl M.A., Religa P., Clasper S., Garvin S., Galter D., Meister B., Ikomi F., Tritsaris K., Dissing S. (2004). PDGF-BB Induces Intratumoral Lymphangiogenesis and Promotes Lymphatic Metastasis. Cancer Cell.

[25] Cao R., Eriksson A., Kubo H., Alitalo K., Cao Y., Thyberg J. (2004). Comparative Evaluation of FGF-2–, VEGF-A–, and VEGF-C–Induced Angiogenesis, Lymphangiogenesis, Vascular Fenestrations, and Permeability. Circ. Res..

[26] Hirakawa S., Kodama S., Kunstfeld R., Kajiya K., Brown L.F., Detmar M. (2005). VEGF-A Induces Tumor and Sentinel Lymph Node Lymphangiogenesis and Promotes Lymphatic Metastasis. J. Exp. Med..

[27] Björndahl M.A., Cao R., Burton J.B., Brakenhielm E., Religa P., Galter D., Wu L., Cao Y. (2005). Vascular Endothelial Growth Factor-A Promotes Peritumoral Lymphangiogenesis and Lymphatic Metastasis. Cancer Res..

[28] Kajiya K., Hirakawa S., Ma B., Drinnenberg I., Detmar M. (2005). Hepatocyte Growth Factor Promotes Lymphatic Vessel Formation and Function. EMBO J..

[29] Morisada T., Oike Y., Yamada Y., Urano T., Akao M., Kubota Y., Maekawa H., Kimura Y., Ohmura M., Miyamoto T. (2005). Angiopoietin-1 Promotes LYVE-1-Positive Lymphatic Vessel Formation. Blood.

[30] Paduch R. (2016). The Role of Lymphangiogenesis and Angiogenesis in Tumor Metastasis. Cell Oncol..

[31] Ucuzian A.A., Greisler H.P. (2007). In Vitro Models of Angiogenesis. World J. Surg..

[32] Morin K.T., Tranquillo R.T. (2013). In Vitro Models of Angiogenesis and Vasculogenesis in Fibrin Gel. Exp. Cell Res..

[33] Norrby K. (2006). In Vivo Models of Angiogenesis. J. Cell. Mol. Med..

[34] Staton C.A., Reed M.W.R., Brown N.J. (2009). A Critical Analysis of Current in Vitro and in Vivo Angiogenesis Assays: Current in Vitro and in Vivo Angiogenesis Assays. Int. J. Exp. Pathol..

[35] Hunter S., Nault B., Ugwuagbo K.C., Maiti S., Majumder M. (2019). Mir526b and Mir655 Promote Tumour Associated Angiogenesis and Lymphangiogenesis in Breast Cancer. Cancers.

[36] Roudsari L.C., West J.L. (2016). Studying the Influence of Angiogenesis in in Vitro Cancer Model Systems. Adv. Drug Deliv. Rev..

[37] Schuermann A., Helker C.S.M., Herzog W. (2014). Angiogenesis in Zebrafish. Semin. Cell Dev. Biol..

[38] Ugwuagbo K.C., Maiti S., Omar A., Hunter S., Nault B., Northam C., Majumder M. (2019). Prostaglandin E2 Promotes Embryonic Vascular Development and Maturation in Zebrafish. Biol. Open.

[39] Doh S.J., Yamakawa M., Santosa S.M., Montana M., Guo K., Sauer J.R., Curran N., Han K.-Y., Yu C., Ema M. (2018). Fluorescent Reporter Transgenic Mice for in Vivo Live Imaging of Angiogenesis and Lymphangiogenesis. Angiogenesis.

[40] Guedez L., Rivera A.M., Salloum R., Miller M.L., Diegmueller J.J., Bungay P.M., Stetler-Stevenson W.G. (2003). Quantitative Assessment of Angiogenic Responses by the Directed in Vivo Angiogenesis Assay. Am. J. Pathol..

[41] Bruyère F., Melen-Lamalle L., Blacher S., Roland G., Thiry M., Moons L., Frankenne F., Carmeliet P., Alitalo K., Libert C. (2008). Modeling Lymphangiogenesis in a Three-Dimensional Culture System. Nat. Methods.

[42] Majumder M., Xin X., Lala P.K. (2013). A Practical and Sensitive Method of Quantitating Lymphangiogenesis in Vivo. Lab. Invest..

[43] Majumder M., Tutunea-Fatan E., Xin X., Rodriguez-Torres M., Torres-Garcia J., Wiebe R., Timoshenko A.V., Bhattacharjee R.N., Chambers A.F., Lala P.K. (2012). Co-Expression of A9β1 Integrin and VEGF-D Confers Lymphatic Metastatic Ability to a Human Breast Cancer Cell Line MDA-MB-468LN. PLoS ONE.

[44] Hosono K., Isonaka R., Kawakami T., Narumiya S., Majima M. (2016). Signaling of Prostaglandin E Receptors, EP3 and EP4 Facilitates Wound Healing and Lymphangiogenesis with Enhanced Recruitment of M2 Macrophages in Mice. PLoS ONE.

[45] Cao R., Lim S., Ji H., Zhang Y., Yang Y., Honek J., Hedlund E.-M., Cao Y. (2011). Mouse Corneal Lymphangiogenesis Model. Nat. Protoc..

[46] Koukourakis M.I. (2005). LYVE-1 Immunohistochemical Assessment of Lymphangiogenesis in Endometrial and Lung Cancer. J. Clin. Pathol..

[47] Kang Y.-J., Mbonye U.R., DeLong C.J., Wada M., Smith W.L. (2007). Regulation of Intracellular Cyclooxygenase Levels by Gene Transcription and Protein Degradation. Prog. Lipid Res..

[48] Yuan C.J., Mandal A.K., Zhang Z., Mukherjee A.B. (2000). Transcriptional Regulation of Cyclooxygenase-2 Gene Expression: Novel Effects of Nonsteroidal Anti-Inflammatory Drugs. Cancer Res..

[49] Teo S.T., Yung Y.C., Herr D.R., Chun J. (2009). Lysophosphatidic Acid in Vascular Development and Disease. IUBMB Life.

[50] Oyesanya R.A., Lee Z.P., Wu J., Chen J., Song Y., Mukherjee A., Dent P., Kordula T., Zhou H., Fang X. (2008). Transcriptional and Post-transcriptional Mechanisms for Lysophosphatidic Acid-induced Cyclooxygenase-2 Expression in Ovarian Cancer Cells. FASEB J..

[51] Xin X., Majumder M., Girish G.V., Mohindra V., Maruyama T., Lala P.K. (2012). Targeting COX-2 and EP4 to Control Tumor Growth, Angiogenesis, Lymphangiogenesis and Metastasis to the Lungs and Lymph Nodes in a Breast Cancer Model. Lab. Invest..

[52] Lala P.K., Nandi P., Majumder M. (2018). Roles of Prostaglandins in Tumor-Associated Lymphangiogenesis with Special Reference to Breast Cancer. Cancer Metastasis Rev..

[53] Majumder M., Nandi P., Omar A., Ugwuagbo K., Lala P. (2018). EP4 as a Therapeutic Target for Aggressive Human Breast Cancer. IJMS.

[54] Pan M.-R., Hou M.-F., Chang H.-C., Hung W.-C. (2008). Cyclooxygenase-2 Up-Regulates CCR7 via EP2/EP4 Receptor Signaling Pathways to Enhance Lymphatic Invasion of Breast Cancer Cells. J. Biol. Chem..

[55] Dimberg A., Bruserud O. (2010). Chemokines in Angiogenesis. The Chemokine System in Experimental and Clinical Hematology.

[56] Wang D., DuBois R.N. (2010). Eicosanoids and Cancer. Nat. Rev. Cancer.

[57] Howe L.R. (2007). Inflammation and Breast Cancer. Cyclooxygenase/Prostaglandin Signaling and Breast Cancer. Breast Cancer Res..

[58] Greenhough A., Smartt H.J.M., Moore A.E., Roberts H.R., Williams A.C., Paraskeva C., Kaidi A. (2009). The COX-2/PGE2 Pathway: Key Roles in the Hallmarks of Cancer and Adaptation to the Tumour Microenvironment. Carcinogenesis.

[59] Tutunea-Fatan E., Majumder M., Xin X., Lala P.K. (2015). The Role of CCL21/CCR7 Chemokine Axis in Breast Cancer-Induced Lymphangiogenesis. Mol. Cancer.

[60] Hanahan D., Weinberg R.A. (2011). Hallmarks of Cancer: The Next Generation. Cell.

[61] Valdés-Rives S.A., González-Arenas A. (2017). Autotaxin-Lysophosphatidic Acid: From Inflammation to Cancer Development. Mediat. Inflamm..

[62] Benesch M.G.K., Tang X., Dewald J., Dong W.-F., Mackey J.R., Hemmings D.G., McMullen T.P.W., Brindley D.N. (2015). Tumor-Induced Inflammation in Mammary Adipose Tissue Stimulates a Vicious Cycle of Autotaxin Expression and Breast Cancer Progression. FASEB J..

[63] Rodriguez Perez C.E., Nie W., Sinnett-Smith J., Rozengurt E., Yoo J. (2011). TNF-α Potentiates Lysophosphatidic Acid-Induced COX-2 Expression via PKD in Human Colonic Myofibroblasts. Am. J. Physiol.-Gastrointest. Liver Physiol..

[64] De Palma M., Biziato D., Petrova T.V. (2017). Microenvironmental Regulation of Tumour Angiogenesis. Nat. Rev. Cancer.

[65] Gervin E., Shin B., Opperman R., Cullen M., Feser R., Maiti S., Majumder M. (2020). Chemically Induced Hypoxia Enhances MiRNA Functions in Breast Cancer. Cancers.

[66] Siemann D.W. (2011). The Unique Characteristics of Tumor Vasculature and Preclinical Evidence for Its Selective Disruption by Tumor-Vascular Disrupting Agents. Cancer Treat. Rev..

[67] Goel S., Duda D.G., Xu L., Munn L.L., Boucher Y., Fukumura D., Jain R.K. (2011). Normalization of the Vasculature for Treatment of Cancer and Other Diseases. Physiol. Rev..

[68] Jayson G.C., Kerbel R., Ellis L.M., Harris A.L. (2016). Antiangiogenic Therapy in Oncology: Current Status and Future Directions. Lancet.

[69] Abdalla A.M.E., Xiao L., Ullah M.W., Yu M., Ouyang C., Yang G. (2018). Current Challenges of Cancer Anti-Angiogenic Therapy and the Promise of Nanotherapeutics. Theranostics.

[70] Rozic J.G., Chakraborty C., Lala P.K. (2001). Cyclooxygenase Inhibitors Retard Murine Mammary Tumor Progression by Reducing Tumor Cell Migration, Invasiveness and Angiogenesis. Int. J. Cancer.

[71] Leek R.D., Lewis C.E., Whitehouse R., Greenall M., Clarkee J., Harris A.L. (1996). Association of Macrophage Infiltration with Angiogenesis and Prognosis in Invasive Breast Carcinoma. Cancer Res..

[72] Majumder M., Xin X., Liu L., Girish G.V., Lala P.K. (2014). Prostaglandin E2 Receptor EP 4 as the Common Target on Cancer Cells and Macrophages to Abolish Angiogenesis, Lymphangiogenesis, Metastasis, and Stem-like Cell Functions. Cancer Sci..

[73] Lin E.Y., Li J.-F., Gnatovskiy L., Deng Y., Zhu L., Grzesik D.A., Qian H., Xue X.-N., Pollard J.W. (2006). Macrophages Regulate the Angiogenic Switch in a Mouse Model of Breast Cancer. Cancer Res..

[74] Vaahtomeri K., Alitalo K. (2020). Lymphatic Vessels in Tumor Dissemination versus Immunotherapy. Cancer Res..

[75] Lyons T.R., O’Brien J., Borges V.F., Conklin M.W., Keely P.J., Eliceiri K.W., Marusyk A., Tan A.-C., Schedin P. (2011). Postpartum Mammary Gland Involution Drives Progression of Ductal Carcinoma in Situ through Collagen and COX-2. Nat. Med..

[76] Farnsworth R.H., Achen M.G., Stacker S.A. (2018). The Evolving Role of Lymphatics in Cancer Metastasis. Curr. Opin. Immunol..

[77] Nandi P., Girish G.V., Majumder M., Xin X., Tutunea-Fatan E., Lala P.K. (2017). PGE2 Promotes Breast Cancer-Associated Lymphangiogenesis by Activation of EP4 Receptor on Lymphatic Endothelial Cells. BMC Cancer.

[78] Majumder M., Landman E., Liu L., Hess D., Lala P.K. (2015). COX-2 Elevates Oncogenic MiR-526b in Breast Cancer by EP4 Activation. Mol. Cancer Res..

[79] Karnezis T., Shayan R., Fox S., Achen M.G., Stacker S.A. (2012). The Connection between Lymphangiogenic Signalling and Prostaglandin Biology: A Missing Link in the Metastatic Pathway. Oncotarget.

[80] Iwata C., Kano M.R., Komuro A., Oka M., Kiyono K., Johansson E., Morishita Y., Yashiro M., Hirakawa K., Kaminishi M. (2007). Inhibition of Cyclooxygenase-2 Suppresses Lymph Node Metastasis via Reduction of Lymphangiogenesis. Cancer Res..

[81] Chan G., Boyle J.O., Yang E.K., Zhang F., Sacks P.G., Shah J.P., Edelstein D., Soslow R.A., Koki A.T., Woerner B.M. (1999). Cyclooxygenase-2 Expression Is up-Regulated in Squamous Cell Carcinoma of the Head and Neck. Cancer Res..

[82] Tsujii M., Kawano S., DuBois R.N. (1997). Cyclooxygenase-2 Expression in Human Colon Cancer Cells Increases Metastatic Potential. Proc. Natl. Acad. Sci. USA.

[83] Parrett M., Harris R., Joarder F., Ross M., Clausen K., Robertson F. (1997). Cyclooxygenase-2 Gene Expression in Human Breast Cancer. Int. J. Oncol..

[84] Lyons T.R., Borges V.F., Betts C.B., Guo Q., Kapoor P., Martinson H.A., Jindal S., Schedin P. (2014). Cyclooxygenase-2–Dependent Lymphangiogenesis Promotes Nodal Metastasis of Postpartum Breast Cancer. J. Clin. Invest..

[85] Tucker O.N., Dannenberg A.J., Yang E.K., Zhang F., Teng L., Daly J.M., Soslow R.A., Masferrer J.L., Woerner B.M., Koki A.T. (1999). Cyclooxygenase-2 Expression Is up-Regulated in Human Pancreatic Cancer. Cancer Res..

[86] Ristimäki A., Sivula A., Lundin J., Lundin M., Salminen T., Haglund C., Joensuu H., Isola J. (2002). Prognostic Significance of Elevated Cyclooxygenase-2 Expression in Breast Cancer. Cancer Res..

[87] Mullen A., Loscher C.E., Roche H.M. (2010). Anti-Inflammatory Effects of EPA and DHA Are Dependent upon Time and Dose-Response Elements Associated with LPS Stimulation in THP-1-Derived Macrophages. J. Nutr. Biochem..

[88] Lee S.A., Kim H.J., Chang K.C., Baek J.C., Park J.K., Shin J.K., Choi W.J., Lee J.H., Paik W.Y. (2009). DHA and EPA Down-Regulate COX-2 Expression through Suppression of NF-ΚB Activity in LPS-Treated Human Umbilical Vein Endothelial Cells. Korean J. Physiol. Pharmacol..

[89] Massaro M., Habib A., Lubrano L., Turco S.D., Lazzerini G., Bourcier T., Weksler B.B., De Caterina R. (2006). The Omega-3 Fatty Acid Docosahexaenoate Attenuates Endothelial Cyclooxygenase-2 Induction through Both NADP(H) Oxidase and PKC Inhibition. Proc. Natl. Acad. Sci. USA.

[90] O’Callaghan G., Houston A. (2015). Prostaglandin E2 and the EP Receptors in Malignancy: Possible Therapeutic Targets?: PGE _2_ Receptors as Targets in Cancer Therapy. Br. J. Pharmacol..

[91] Sugimoto Y., Narumiya S. (2007). Prostaglandin E Receptors. J. Biol. Chem..

[92] Markovič T., Jakopin Ž., Dolenc M.S., Mlinarič-Raščan I. (2017). Structural Features of Subtype-Selective EP Receptor Modulators. Drug Discov. Today.

[93] Hida T., Yatabe Y., Achiwa H., Muramatsu H., Kozaki K., Nakamura S., Ogawa M., Mitsudomi T., Sugiura T., Takahashi T. (1998). Increased Expression of Cyclooxygenase 2 Occurs Frequently in Human Lung Cancers, Specifically in Adenocarcinomas. Cancer Res..

[94] Liu C.H., Chang S.-H., Narko K., Trifan O.C., Wu M.-T., Smith E., Haudenschild C., Lane T.F., Hla T. (2001). Overexpression of Cyclooxygenase-2 Is Sufficient to Induce Tumorigenesis in Transgenic Mice. J. Biol. Chem..

[95] Chulada P.C., Thompson M.B., Mahler J.F., Doyle C.M., Gaul B.W., Lee C., Tiano H.F., Morham S.G., Smithies O., Langenbach R. (2000). Genetic Disruption of Ptgs-1, as Well as Ptgs-2, Reduces Intestinal Tumorigenesis in Min Mice. Cancer Res..

[96] Harris R.E. (2003). COX-2 Blockade in Cancer Prevention and Therapy.

[97] Harris R.E., Chlebowski R.T., Jackson R.D., Frid D.J., Ascenseo J.L., Anderson G., Loar A., Rodabough R.J., White E., McTiernan A. (2003). Breast Cancer and Nonsteroidal Anti-Inflammatory Drugs: Prospective Results from the Women’s Health Initiative. Cancer Res..

[98] Gupta A.K., Schoen R.E. (2009). Aberrant Crypt Foci: Are They Intermediate Endpoints of Colon Carcinogenesis in Humans?. Curr. Opin. Gastroenterol..

[99] Howe L.R., Dannenberg A.J. (2003). COX-2 Inhibitors for the Prevention of Breast Cancer. J. Mammary Gland Biol. Neoplasia.

[100] Sharpe C.R., Collet J.-P., McNutt M., Belzile E., Boivin J.-F., Hanley J.A. (2000). Nested Case–Control Study of the Effects of Non-Steroidal Anti-Inflammatory Drugs on Breast Cancer Risk and Stage. Br. J. Cancer.

[101] Oshima M., Dinchuk J.E., Kargman S.L., Oshima H., Hancock B., Kwong E., Trzaskos J.M., Evans J.F., Taketo M.M. (1996). Suppression of Intestinal Polyposis in ApcΔ716 Knockout Mice by Inhibition of Cyclooxygenase 2 (COX-2). Cell.

[102] Parhar R.S., Lala P.K. (1985). Changes in the Host Natural Killer Cell Population in Mice during Tumor Development. Cell. Immunol..

[103] Lala P.K., Parhar R.S., Singh P. (1986). Indomethacin Therapy Abrogates the Prostaglandin-Mediated Suppression of Natural Killer Activity in Tumor-Bearing Mice and Prevents Tumor Metastasis. Cell. Immunol..

[104] Lala P.K., Al-Mutter N., Orucevic A. (1997). Effects of Chronic Indomethacin Therapy on the Development and Progression of Spontaneous Mammary Tumors in C3H/HEJ Mice. Int. J. Cancer.

[105] Kundu N., Ma X., Holt D., Goloubeva O., Ostrand-Rosenberg S., Fulton A.M. (2009). Antagonism of the Prostaglandin E Receptor EP4 Inhibits Metastasis and Enhances NK Function. Breast Cancer Res. Treat..

[106] Timoshenko A.V., Xu G., Chakrabarti S., Lala P.K., Chakraborty C. (2003). Role of Prostaglandin E2 Receptors in Migration of Murine and Human Breast Cancer Cells. Exp. Cell Res..

[107] Timoshenko A.V., Lala P.K., Chakraborty C. (2004). PGE2-Mediated Upregulation of INOS in Murine Breast Cancer Cells through the Activation of EP4 Receptors. Int. J. Cancer.

[108] Xu L., Stevens J., Hilton M.B., Seaman S., Conrads T.P., Veenstra T.D., Logsdon D., Morris H., Swing D.A., Patel N.L. (2014). COX-2 Inhibition Potentiates Antiangiogenic Cancer Therapy and Prevents Metastasis in Preclinical Models. Sci. Transl. Med..

[109] Timoshenko A.V., Chakraborty C., Wagner G.F., Lala P.K. (2006). COX-2-Mediated Stimulation of the Lymphangiogenic Factor VEGF-C in Human Breast Cancer. Br. J. Cancer.

[110] Hiken J.F., McDonald J.I., Decker K.F., Sanchez C., Hoog J., VanderKraats N.D., Jung K.L., Akinhanmi M., Rois L.E., Ellis M.J. (2017). Epigenetic Activation of the Prostaglandin Receptor EP4 Promotes Resistance to Endocrine Therapy for Breast Cancer. Oncogene.

[111] Holt D., Ma X., Kundu N., Fulton A. (2011). Prostaglandin E2 (PGE2) Suppresses Natural Killer Cell Function Primarily through the PGE2 Receptor EP4. Cancer Immunol. Immunother..

[112] Ma X., Holt D., Kundu N., Reader J., Goloubeva O., Take Y., Fulton A.M. (2013). A Prostaglandin E (PGE) Receptor EP4 Antagonist Protects Natural Killer Cells from PGE _2_ -Mediated Immunosuppression and Inhibits Breast Cancer Metastasis. OncoImmunology.

[113] Okano M., Sugata Y., Fujiwara T., Matsumoto R., Nishibori M., Shimizu K., Maeda M., Kimura Y., Kariya S., Hattori H. (2006). E Prostanoid 2 (EP2)/EP4-Mediated Suppression of Antigen-Specific Human T-Cell Responses by Prostaglandin E2. Immunology.

[114] Albu D.I., Wang Z., Wu J., Huang K., Li W., Liu D., Kuznetsov G., Chen Q., Bao X., Woodall-Jappe M. Abstract 275: ER-886046, an Antagonist of PGE2 Receptor Type-4, Induces an Effective Antitumor Immune Response in Mice by Attenuating Intratumoral MDSCs and TAMs. Proceedings of the AACR 106th Annual Meeting 2015.

[115] Kundu N., Ma X., Kochel T., Goloubeva O., Staats P., Thompson K., Martin S., Reader J., Take Y., Collin P. (2014). Prostaglandin E Receptor EP4 Is a Therapeutic Target in Breast Cancer Cells with Stem-like Properties. Breast Cancer Res. Treat..

[116] Wicha M.S., Liu S., Dontu G. (2006). Cancer Stem Cells: An Old Idea—A Paradigm Shift. Cancer Res..

[117] Tysnes B.B. (2010). Tumor-Initiating and -Propagating Cells: Cells That We Would to Identify and Control. Neoplasia.

[118] Li X., Lewis M.T., Huang J., Gutierrez C., Osborne C.K., Wu M.-F., Hilsenbeck S.G., Pavlick A., Zhang X., Chamness G.C. (2008). Intrinsic Resistance of Tumorigenic Breast Cancer Cells to Chemotherapy. JNCI J. Natl. Cancer Inst..

[119] Visvader J.E., Lindeman G.J. (2012). Cancer Stem Cells: Current Status and Evolving Complexities. Cell Stem Cell.

[120] FitzGerald G.A. (2004). Coxibs and Cardiovascular Disease. N. Engl. J. Med..

[121] Graham D.J. (2006). COX-2 Inhibitors, Other NSAIDs, and Cardiovascular Risk: The Seduction of Common Sense. JAMA.

[122] Fujino H., Xu W., Regan J.W. (2003). Prostaglandin E _2_ Induced Functional Expression of Early Growth Response Factor-1 by EP _4_, but Not EP _2_, Prostanoid Receptors via the Phosphatidylinositol 3-Kinase and Extracellular Signal-Regulated Kinases. J. Biol. Chem..

[123] Xiao C.-Y., Hara A., Yuhki K., Fujino T., Ma H., Okada Y., Takahata O., Yamada T., Murata T., Narumiya S. (2001). Roles of Prostaglandin I _2_ and Thromboxane A _2_ in Cardiac Ischemia-Reperfusion Injury: A Study Using Mice Lacking Their Respective Receptors. Circulation.

[124] Martin M., Meyer-Kirchrath J., Kaber G., Jacoby C., Flögel U., Schrader J., Rüther U., Schrör K., Hohlfeld T. (2005). Cardiospecific Overexpression of the Prostaglandin EP _3_ Receptor Attenuates Ischemia-Induced Myocardial Injury. Circulation.

[125] Thiemermann C., Zacharowski K. (2000). Selective Activation of E-Type Prostanoid3-Receptors Reduces Myocardial Infarct Size. Pharmacol. Ther..

[126] Mediratta K., El-Sahli S., D’Costa V., Wang L. (2020). Current Progresses and Challenges of Immunotherapy in Triple-Negative Breast Cancer. Cancers.

[127] Mosalpuria K., Hall C., Krishnamurthy S., Lodhi A., Hallman D.M., Baraniuk M.S., Bhattacharyya A., Lucci A. (2014). Cyclooxygenase-2 Expression in Non-Metastatic Triple-Negative Breast Cancer Patients. Mol. Clin. Oncol..

[128] Kochel T.J., Goloubeva O.G., Fulton A.M. (2016). Upregulation of Cyclooxygenase-2/Prostaglandin E_2_ (COX-2/PGE_2_) Pathway Member Multiple Drug Resistance-Associated Protein 4 (MRP4) and Downregulation of Prostaglandin Transporter (PGT) and 15-Prostaglandin Dehydrogenase (15-PGDH) in Triple-Negative Breast Cancer. Breast Cancer.

[129] Williams C.S., Tsujii M., Reese J., Dey S.K., DuBois R.N. (2000). Host Cyclooxygenase-2 Modulates Carcinoma Growth. J. Clin. Invest..

[130] Bhattacharjee R.N., Timoshenko A.V., Cai J., Lala P.K. (2010). Relationship between Cyclooxygenase-2 and Human Epidermal Growth Factor Receptor 2 in Vascular Endothelial Growth Factor C up-Regulation and Lymphangiogenesis in Human Breast Cancer. Cancer Sci..

[131] Majumder M., Dunn L., Liu L., Hasan A., Vincent K., Brackstone M., Hess D., Lala P.K. (2018). COX-2 Induces Oncogenic Micro RNA MiR655 in Human Breast Cancer. Sci. Rep..

[132] Tordjman J., Majumder M., Amiri M., Hasan A., Hess D., Lala P.K. (2019). Tumor Suppressor Role of Cytoplasmic Polyadenylation Element Binding Protein 2 (CPEB2) in Human Mammary Epithelial Cells. BMC Cancer.

[133] Chen P.-J., Huang Y.-S. (2012). CPEB2-EEF2 Interaction Impedes HIF-1α RNA Translation: Translation Control at Elongation. EMBO J..

[134] Li N.-N., Meng X.-S., Bao Y.-R., Wang S., Li T.-J. (2018). Evidence for the Involvement of COX-2/VEGF and PTEN/Pl3K/AKT Pathway the Mechanism of Oroxin B Treated Liver Cancer. Pharmacogn. Mag..

[135] Forsythe J.A., Jiang B.H., Iyer N.V., Agani F., Leung S.W., Koos R.D., Semenza G.L. (1996). Activation of Vascular Endothelial Growth Factor Gene Transcription by Hypoxia-Inducible Factor 1. Mol. Cell. Biol..

[136] Hirota K., Semenza G.L. (2006). Regulation of Angiogenesis by Hypoxia-Inducible Factor 1. Crit. Rev. Oncol./Hematol..

[137] Howe L.R., Chang S.-H., Tolle K.C., Dillon R., Young L.J.T., Cardiff R.D., Newman R.A., Yang P., Thaler H.T., Muller W.J. (2005). HER2/Neu-Induced Mammary Tumorigenesis and Angiogenesis Are Reduced in Cyclooxygenase-2 Knockout Mice. Cancer Res..

[138] Kundu N., Fulton A.M. (2002). Selective Cyclooxygenase (COX)-1 or COX-2 Inhibitors Control Metastatic Disease in a Murine Model of Breast Cancer. Cancer Res..

[139] Ma X., Kundu N., Rifat S., Walser T., Fulton A.M. (2006). Prostaglandin E Receptor EP4 Antagonism Inhibits Breast Cancer Metastasis. Cancer Res..

[140] Albu D.I., Wang Z., Huang K.-C., Wu J., Twine N., Leacu S., Ingersoll C., Parent L., Lee W., Liu D. (2017). EP4 Antagonism by E7046 Diminishes Myeloid Immunosuppression and Synergizes with Treg-Reducing IL-2-Diphtheria Toxin Fusion Protein in Restoring Anti-Tumor Immunity. OncoImmunology.

[141] Lala P.K., Elkashab M., Kerbel R.S., Parhar R.S. (1990). Cure of Human Melanoma Lung Metastases in Nude Mice with Chronic Indomethacin Therapy Combined with Multiple Rounds of IL-2: Characteristics of Killer Cells Generated in Situ. Int. Immunol..

[142] Singh B., Berry J.A., Shoher A., Ayers G.D., Wei C., Lucci A. (2007). COX-2 Involvement in Breast Cancer Metastasis to Bone. Oncogene.

[143] Majumder M., Xin X., Liu L., Tutunea-Fatan E., Rodriguez-Torres M., Vincent K., Postovit L.-M., Hess D., Lala P.K. (2016). COX-2 Induces Breast Cancer Stem Cells via EP4/PI3K/AKT/NOTCH/WNT Axis: Targeting EP4 to Abrogate Breast Cancer Stem Cells. Stem Cells.

[144] Mestas J., Hughes C.C.W. (2004). Of Mice and Not Men: Differences between Mouse and Human Immunology. J. Immunol..

[145] Brown M.E., Zhou Y., McIntosh B.E., Norman I.G., Lou H.E., Biermann M., Sullivan J.A., Kamp T.J., Thomson J.A., Anagnostopoulos P.V. (2018). A Humanized Mouse Model Generated Using Surplus Neonatal Tissue. Stem Cell Rep..

[146] Lan P., Tonomura N., Shimizu A., Wang S., Yang Y.-G. (2006). Reconstitution of a Functional Human Immune System in Immunodeficient Mice through Combined Human Fetal Thymus/Liver and CD34+ Cell Transplantation. Blood.

[147] Dobrolecki L.E., Airhart S.D., Alferez D.G., Aparicio S., Behbod F., Bentires-Alj M., Brisken C., Bult C.J., Cai S., Clarke R.B. (2016). Patient-Derived Xenograft (PDX) Models in Basic and Translational Breast Cancer Research. Cancer Metastasis Rev..

[148] Wang M., Yao L.-C., Cheng M., Cai D., Martinek J., Pan C.-X., Shi W., Ma A.-H., De Vere White R.W., Airhart S. (2018). Humanized Mice in Studying Efficacy and Mechanisms of PD-1-Targeted Cancer Immunotherapy. FASEB J..

[149] Bao X., Albu D., Huang K.-C., Wu J., Twine N., Nomoto K., Woodall-Jappe M. (2015). Combination of EP4 Antagonist and Checkpoint Inhibitors Promotes Anti-Tumor Effector T Cells in Preclinical Tumor Models. J. Immunother. Cancer.

[150] Planes-Laine G., Rochigneux P., Bertucci F., Chrétien A.S., Viens P., Sabatier R., Gonçalves A. (2019). PD-1/PD-L1 Targeting in Breast Cancer: The First Clinical Evidences Are Emerging. A Literature Review. Cancers.

[151] Marra A., Viale G., Curigliano G. (2019). Recent Advances in Triple Negative Breast Cancer: The Immunotherapy Era. BMC Med..

[152] Schmid P., Cortes J., Pusztai L., McArthur H., Kümmel S., Bergh J., Denkert C., Park Y.H., Hui R., Harbeck N. (2020). Pembrolizumab for Early Triple-Negative Breast Cancer. N. Engl. J. Med..

[153] Ching M.M., Reader J., Fulton A.M. (2020). Eicosanoids in Cancer: Prostaglandin E2 Receptor 4 in Cancer Therapeutics and Immunotherapy. Front. Pharmacol..

[154] Take Y., Koizumi S., Nagahisa A. (2020). Prostaglandin E Receptor 4 Antagonist in Cancer Immunotherapy: Mechanisms of Action. Front. Immunol..

[155] Okumura Y., Yamagishi T., Nukui S., Nakao K. (2017). Discovery of AAT-008, a Novel, Potent, and Selective Prostaglandin EP4 Receptor Antagonist. Bioorganic Med. Chem. Lett..

[156] Hong D.S., Parikh A., Shapiro G.I., Varga A., Naing A., Meric-Bernstam F., Ataman Ö., Reyderman L., Binder T.A., Ren M. (2020). First-in-Human Phase I Study of Immunomodulatory E7046, an Antagonist of PGE _2_ -Receptor E-Type 4 (EP4), in Patients with Advanced Cancers. J. Immunother. Cancer.

